# Electrodermal Activity (EDA) Morphologies and Prediction of Engagement with Simple Moving Average Crossover: A Mixed-Method Study

**DOI:** 10.3390/s24144565

**Published:** 2024-07-14

**Authors:** Kishore Kumar Nandipati, Sonika Pal, Ritayan Mitra

**Affiliations:** Educational Technology, Indian Institute of Technology Bombay, Mumbai 400076, India; kishorenandipati975@gmail.com (K.K.N.); sonika_pal@iitb.ac.in (S.P.)

**Keywords:** electrodermal activity (EDA), galvanic skin response (GSR), moving average crossover, EDA morphologies, engagement, education

## Abstract

Electrodermal Activity (EDA), which primarily indicates arousal through sympathetic nervous system activity, serves as a tool to measure constructs like engagement, cognitive load, performance, and stress. Despite its potential, empirical studies have often yielded mixed results and found it of limited use. To better understand EDA, we conducted a mixed-methods study in which quantitative EDA profiles and survey data were investigated using qualitative interviews. This study furnishes an EDA dataset measuring the engagement levels of seven participants who watched three videos for 4–10 min. The subsequent interviews revealed five EDA morphologies with varying short-term signatures and long-term trends. We used this dataset to demonstrate the moving average crossover, a novel metric for EDA analysis, in predicting engagement–disengagement dynamics in such data. Our contributions include the creation of the detailed dataset, comprising EDA profiles annotated with qualitative data, the identification of five distinct EDA morphologies, and the proposition of the moving average crossover as an indicator of the beginning of engagement or disengagement in an individual.

## 1. Introduction and Literature Review

Electrodermal Activity (EDA), also known as the galvanic skin response (GSR), has emerged as a tool for exploring the multifaceted cognitive and affective nature of learning processes. Electrodermal activity indicates sympathetic autonomic nervous system activity, exhibiting minimal susceptibility to parasympathetic influences [1]. This characteristic underscores its robustness as an indicator of sympathetic nervous system activity, thereby mitigating concerns regarding inauthentic participation [2,3]. While EDA primarily measures arousal [4,5,6,7,8,9,10,11,12], its widespread utilization within educational contexts extends to the measurement of variables such as cognitive load [13,14,15,16,17,18,19,20], performance [21,22], stress [23,24,25,26,27], and engagement [28,29,30,31,32,33,34,35,36,37,38,39].

Despite its potential, the application of EDA in educational research has faced challenges, and several studies have found it to be of limited use [40,41,42,43,44]. Machine learning models based on multimodal sensor data often discard EDA features as effective predictors of relevant constructs [45].

The ambiguity and uncertainty around EDA data in the literature can be partly ascribed to the intricate and/or multidimensional nature of the underlying constructs, particularly in learning scenarios. Since arousal is the primary construct measured, disentangling the underlying factors that can ‘arouse’ can be challenging. The other reason is that we have only looked at EDA values from a narrow methodological standpoint. As typical in studies with any sensor data, EDA studies only look to measure and use a quantitative estimate of arousal, which has to be attributed to one or multiple constructs of interest based on research questions and task design. All efforts in the research community have so far been to measure and process this signal more accurately, which has its methodological challenges [46]. More importantly, however, the questions in the community have been most often answered by a correlation between relevant constructs and the arousal data. Therefore, the challenge lies in understanding EDA signals’ accurate import and variability and their correct interpretation [47]. Consensus among researchers is crucial for improving the comparability, generalizability, and replicability of findings, but quantitative correlative studies have not established meaningful causal relationships [48]. Therein lies a more profound gap in the EDA literature: we seem to expect EDA to correlate with arousal. Still, because we lack a proper understanding of its mechanics, we discard the data as often as we use them.

As mentioned above, educational research has often used mixed methods to overcome challenges [49,50]. While a quantitative perspective is beneficial, qualitative data usually provide a more thorough understanding of the causality behind the data [51]. Qualitative education research, guided by the interpretivist paradigm, delves into the complex and culturally influenced nature of learning. It focuses on understanding each learner’s unique experiences, recognizing that these cannot be fully encapsulated by standard statistical models. Together, they form an entirely separate methodological paradigm, known as mixed methods, that proponents argue to be more than the sum of its parts. This approach involves carrying out quantitative and qualitative research simultaneously or in sequence, allowing researchers to balance or emphasize one type over another based on the study’s aims. Mixed-methods research explores a single phenomenon through these dual lenses, aiming for triangulation to integrate and enhance findings. Furthermore, this method facilitates the refinement and clarification of results by incorporating insights from one method with data from another [52].

It has been demonstrated that a mixed-methods approach, incorporating quantitative data such as electrodermal activity (EDA) measurements and survey responses and qualitative data from classroom observations and interviews, can provide comprehensive insights [53]. Subsequently, a sequential strategy, known as “QUAN→qual”, was applied, beginning with the collection and analysis of quantitative data followed by the gathering and examination of qualitative data to enhance the study’s findings. This integration of quantitative and qualitative data enabled effective triangulation, leading to robust and well-supported conclusions regarding the physiological characterization of student engagement. Similarly, a convergent mixed-methods design was employed in another study to analyze the cognitive load and student engagement, integrating both quantitative EDA and qualitative behavioral data. Initially, data were collected simultaneously yet analyzed independently to capture real-time responses during educational tasks. This was followed by synthesizing these data to identify patterns that could explain variations in engagement and cognitive load [54]. This methodological approach ensured a balanced interpretation, leveraging the strengths of both research paradigms.

We seek to further such lines of inquiry by creating an EDA dataset primed to measure engagement, with secondary interview information attached. We had seven participants watch three educational and related media to generate twenty-one EDA profiles. Subsequently, we interviewed the participants independently regarding their thoughts and feelings while watching the videos. We also gave them a short survey to capture their self-reported engagement levels. We interpreted the EDA profiles with the interview and survey data. A careful analysis of the EDA data revealed five morphologies. We witnessed a critical interplay of short-term arousal signatures and long-term trends in all EDA profiles, which account for participant engagement or disengagement trends as revealed through the qualitative interviews. 

Based on the insight gathered from the above analysis, we introduce a contextually novel approach to analyzing EDA by leveraging moving average crossovers. This technique is popular in financial data analysis for spotting trend reversals in the price of any security. Our argument, supported by data, is that engagement and disengagement may show behavior very similar to the bearish and bullish trends observed in the financial markets and, therefore, should be amenable to similar analysis. This technique does not require baseline measurements, offering more flexibility to EDA analysis.

To summarize, this study contributes to the advancement of the understanding and application of EDA in three ways. First, it provides a documented dataset of EDA morphologies with independent secondary qualitative data for researchers to use (see Supplementary Material, Sections SI and SII). Second, it establishes five major types of EDA morphologies. Third, we propose a novel metric, the moving average crossover, for EDA analysis and recommend its future integration into ML models or Intelligent Tutoring Systems (ITS).

## 2. Methodology

We employed a mixed-method research design, also known as triangulation. This approach combines qualitative and quantitative data to arrive at any conclusion. In the quantitative phase, EDA was measured when participants watched the videos. A survey form asked them to rate their cognitive and affective engagement and answer questions such as, “What was the most interesting part of the video? What did you learn from the video?” The EDA and the survey responses allowed us to mark specific sections of the video to help us understand the learner’s engagement while watching the video. Not all segments of an EDA profile would be interpretable, but some, showing peaks and/or having specific mentions in the survey questions, were expected to be more revealing. Following this step, we conducted stimulated recall interviews, where participants only watched video segments we identified in the previous step. The interviewer asked focused questions about their engagement (or disengagement) without showing the actual EDA data. The qualitative insights from these interviews were then systematically integrated with the quantitative EDA profiles, leading to the generation of an annotated EDA dataset (see Section 3.2).

### 2.1. Participants

Seven participants (mean age of 27 ± 5 years, comprising four males and three females) from a prominent technical institute in India were involved. The researcher used convenience sampling to select the participants and obtained informed consent from each after explaining the study details. The experiment adhered to the ethical guidelines set by the Institute Ethics Committee. To protect the privacy of the participants, their identities are kept confidential. Only aggregated results are reported, and any analysis that identified individual participants was conducted in a way that did not reveal their identities.

### 2.2. Equipment and Software

**Figure 1:**
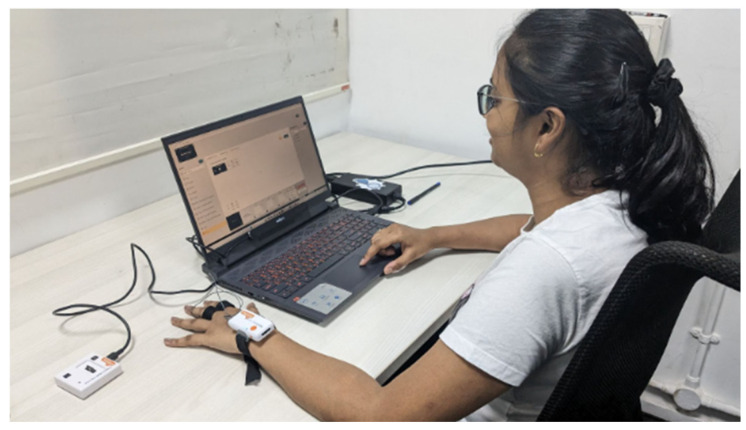
Overview of the experimental settings.

For this study, EDA responses were collected using sensors with a Shimmer3 CD70 GSR+ unit to gather skin conductivity data from participants. The Shimmer3 GSR suit includes a sensor, two electrodes, and a strap. The electrodes are worn on the index and middle fingers of the participant’s non-dominant hand (mostly on the left hand), and the device is secured to the wrist with a strap. The system records data at a frequency of 128 Hz (Figure 1). iMotions software (version 9.3.0) was used for data collection, and Python libraries were used for data analysis.

### 2.3. Video Selection Criteria

Participants were instructed to watch three videos in a fixed sequence in this study. The videos were all related to relativity and black holes, a popular science topic of considerable interest. The first video, sourced from YouTube, titled “Special Theory of Relativity by Andrey K”, was 5 min and 33 s long and briefly discussed Albert Einstein’s special theory of relativity. Following this, another YouTube video, “Travel INSIDE a Black Hole by Vsauce”, played for 9 min and 55 s. This video explores what would happen if one travels into a black hole. The third and final video was taken from the movie “Interstellar”, directed by Christopher Nolan, and this scene features a perilous mission on a water-covered planet called Miller planet near a black hole. The duration of the video was 4 min and 51 s, and the snippet involved discussions on black holes and time dilation. The selected videos were also expected to generate different levels of engagement on similar topics due to their varying presentation styles and content depth. The first video introduces the special theory of relativity using a didactic, traditional lecture style with the instructor standing before a whiteboard and reading through lines of text on it. The second video, with its longer duration and more detailed exploration of traveling into a black hole, uses animation and poses interesting questions along the way; the third video is of a dramatic scene from the movie “Interstellar”, which offers the most visually engaging and emotionally charged representation of the concepts. These differences in presentation and context were intended to elicit engagement in the broadest sense.

### 2.4. Experimental Procedure

Throughout the experiment, participants were equipped with EDA sensors to collect physiological data simultaneously. Each participant was seated comfortably in front of a 15.6-inch laptop with a 1920 × 1080 pixels screen resolution.

Each video started with a blank screen, followed by instructions for 30 s, where the participants were briefed about the video and other basic instructions. After that, there was a 60 s interlude with a gray screen to measure baseline EDA, and subsequently, the video was played. After the video, an “End Slide” was shown for approximately six seconds. 

**Figure 2:**
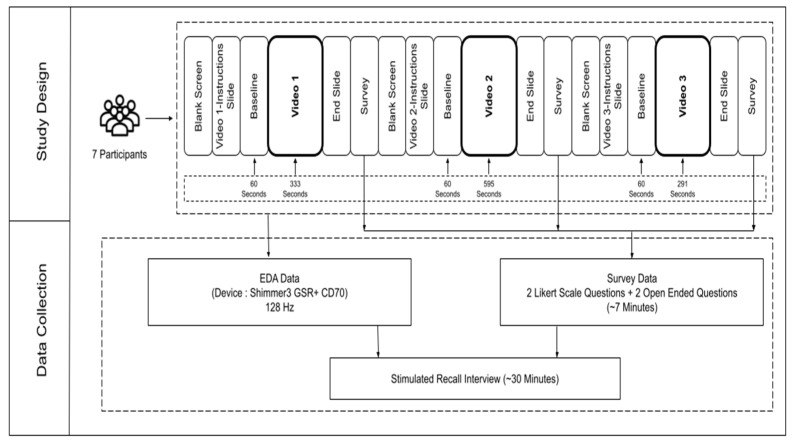
An overview of the study design.

After watching each video, participants were asked to fill out a four-question survey form. Two of these questions measured cognitive and affective engagement levels using a seven-point Likert scale ranging from 1 (Not Engaged) to 7 (Highly Engaged). The other two questions were open-ended and aimed at identifying the most engaging parts of the video and its critical learning takeaways [Supplementary Material, Section SIII]. The survey aimed to gauge the engagement level and moments of engagement in the video to help with the subsequent interview process. The session concluded with participants participating in a stimulated recall interview (Figure 2).

A total of 21 EDA curves could be constructed from the seven participants who watched the three videos. The interview took place right after the experiment. The participants were given a short break, during which the interviewer analyzed the EDA and survey data to create a semi-structured interview protocol for the participant.

**Figure 3:**
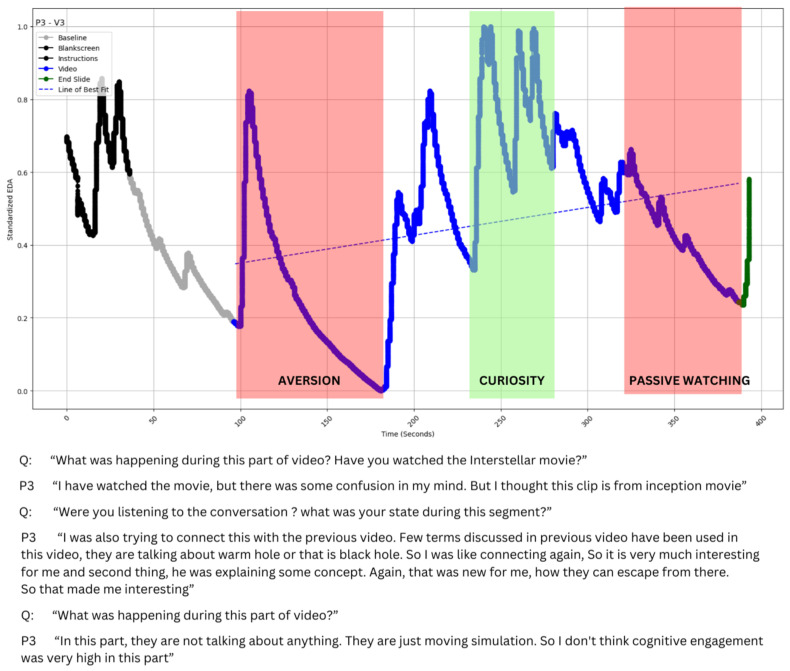
Alignment trends observed in EDA data and insights observed from interview excerpts.

A stimulated interview protocol was employed, where the participant was presented with video segments corresponding to observed trends in Electrodermal Activity (EDA) signals as noted by the interviewer. For example, as illustrated in Figure 3 (103 s to 180 s), the researcher observed a sharp decline in EDA. When asked about their state during this segment, the participant mentioned experiencing confusion because the participant got confused. After all, they thought it was from a different movie. Similarly, when asked about their state between 235 and 260 s, the participant indicated that they attempted to connect the terminologies used in this video with the preceding video, finding new concepts interesting. This way, we could establish two independent data sources—EDA and interviews.

## 3. Data Analysis

### 3.1. EDA Data Cleaning and Pre-Processing

The EDA data were cleaned with a 1 s moving average window to minimize noise and artifacts like body gestures. Following this, preprocessing steps, including normalization ((x − min)/(max − min)), were undertaken to account for individual participant differences. The cleaned and processed EDA data were then visualized, as shown in Figure 3, laying the groundwork for in-depth qualitative interviews to uncover the participants’ detailed experiences.

### 3.2. Interview Analysis

To analyze the stimulated recall interviews, the audio recordings were initially transcribed using an open-source Python code provided by Assembly AI (https://www.assemblyai.com/) accessed on 13 April 2024. The accuracy of the transcriptions was verified by cross-referencing them with the original audio recordings, and they were found to be accurate.

We used an inductive coding method. Initially, open codes were generated from the transcribed data. The data were then organized under sub-codes, such as disconnection, aversion, passive watching, curiosity, recall, and attention [55,56]. The final stage led to development of major themes, such as disengagement and engagement, based on the sub-codes. A part of the comprehensive codebook was subsequently developed (see Supplementary Material, Section SII) to document these findings, and some examples from the codebook are illustrated in Table 1.

Based on the codebook, the first and the second authors coded 25% of the interview data together to ensure complete agreement through discussion, after which the first author coded all the remaining interviews. After completing the coding, the researchers attempted to triangulate the participant trends in the EDA data. It was observed that there was a significant alignment between the interview data and the trends observed in the EDA data (Figure 3).

## 4. Result

### 4.1. Correlation between Survey Data and EDA Metrics (Slope and Mean EDA)

The mean EDA values were corrected for baseline by subtracting the latter. The mean EDA values were investigated for correlation with the survey data that collected cognitive and affective engagement separately on a 7-point Likert scale. Mean EDA and cognitive engagement indicated a correlation that barely missed significance (r (19) = 0.416, *p* = 0.061). However, no correlation was observed between the mean EDA and affective engagement (r (19) = 0.0197, *p* = 0.933). 

**Figure 4:**
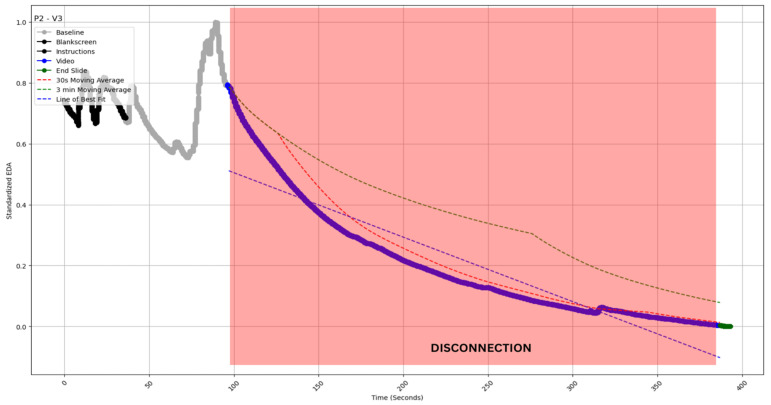
EDA profile of participant 2 watching video 3 (P2-V3 of inset). The profile exhibits a gradual decline between 99 and 385 s. The profile is superimposed (red shaded area) with independently coded interview data, which revealed “disconnection“ during this time period. The blue straight dashed line indicates the slope of EDA. The red and green curved dashed lines indicate the 30 s and 3 min moving averages of the EDA (see Section 5).

**Figure 5:**
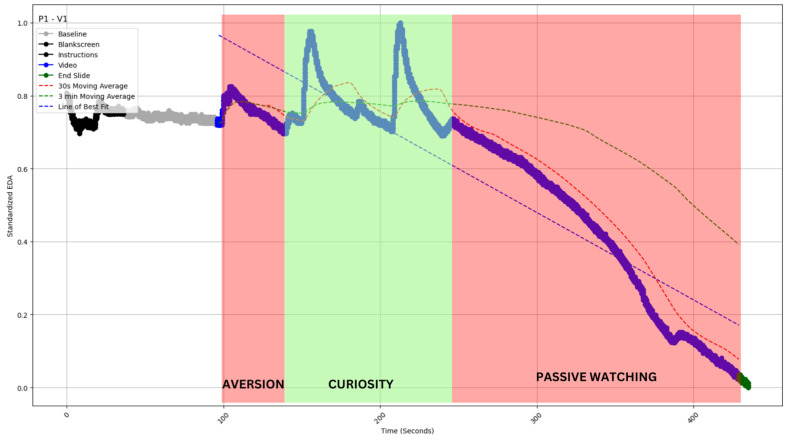
EDA profile of profile of participant 1 watching video 1 (P1-V1 of inset). The profile gradually declines between 105 and 137 s and between 245 and 425 s. The profile is superimposed (red shaded area) with independently coded interview data, which revealed “aversion” and “passive watching” for this period. The profile exhibits a surge between 150 and 213 s. The profile is superimposed (green shaded area) with independently coded interview data, which revealed “curiosity” for this period. The blue straight dashed line indicates the slope of EDA. The red and green curved dashed lines indicate the 30 s and 3 min moving averages of the EDA (see Section 5).

**Figure 6:**
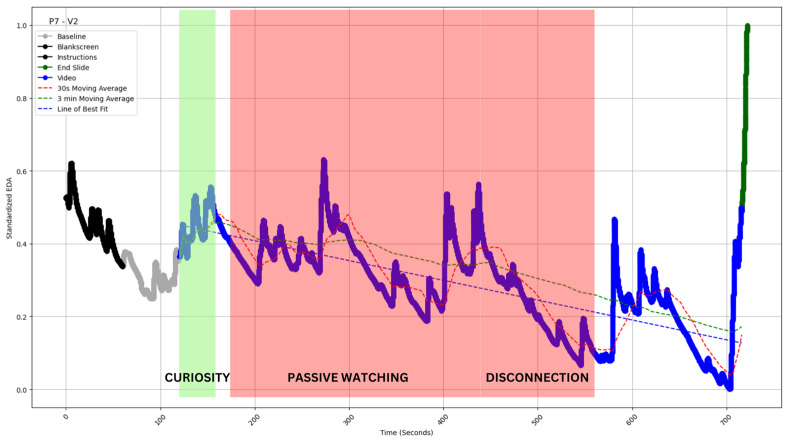
EDA profile of participant 7 watching video 2 (P7-V2 of inset). The profile exhibits a surge between 120 and 155 s. The profile is superimposed (green shaded area) with independently coded interview data, which revealed “curiosity” for this time period. The profile exhibits a decline with multiple peaks between 160 and 560 s. The profile is superimposed (red shaded area) with independently coded interview data, which revealed “passive watching” and “disconnection” for this time period. The blue straight dashed line indicates the slope of EDA. The red and green curved dashed lines indicate the 30 s and 3 min moving averages of the EDA (see Section 5).

**Figure 7:**
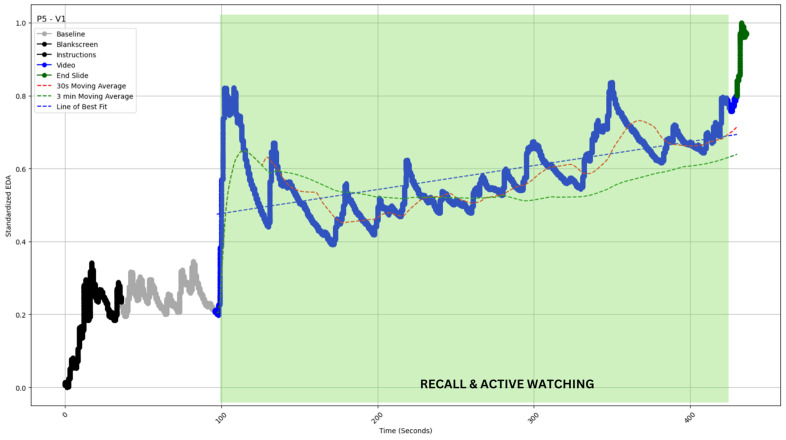
EDA profile of participant 5 watching video 1 (P5-V1 of inset). The profile exhibits a continuous surge with multiple peaks between 97 and 430 s. The profile is superimposed (green shaded area) with independently coded interview data, which revealed “recall” and “active watching” for this time period. The blue straight dashed line indicates the slope of EDA. The red and green curved dashed lines indicate the 30 s and 3 min moving averages of the EDA (see Section 5).

**Figure 8:**
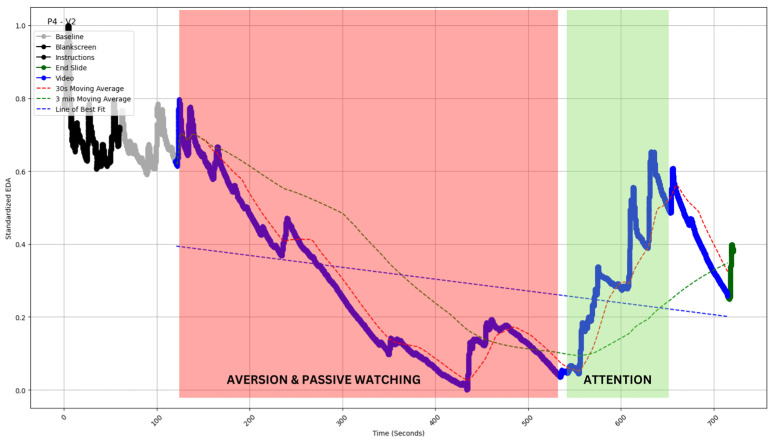
EDA profile of participant 4 watching video 2 (P4-V2 of inset). The profile exhibits a decline with a few peaks between 125 and 430 s. The profile is superimposed (red shaded area) with independently coded interview data, which revealed “aversion” and “passive watching” for this time period. The profile also exhibits a surge with few peaks between 550 and 650 s. The profile is superimposed (green shaded area) with independently coded interview data, which revealed “attention” for this time period. The blue straight dashed line indicates the slope of EDA. The red and green curved dashed lines indicate the 30 s and 3 min moving averages of the EDA (see Section 5).

The slope of the best-fit line for the EDA values (straight dotted lines in Figure 4, Figure 5, Figure 6, Figure 7 and Figure 8) during video observation showed no significant correlation with either cognitive engagement (r (19) = 0.338, *p* = 0.134) or affective engagement (r (19) = 0.344, *p* = 0.127).

### 4.2. Characteristics of EDA Time Series

All twenty-one EDA time series were visually scrutinized and categorized into five distinct categories, focusing on two primary characteristics: variability, which refers to local fluctuations in the signal, and trend, which indicates the overall direction (either upslope or downslope) of the signal. This approach allowed us to group the profiles based on their distinct patterns, providing a structured framework for understanding EDA morphologies.

Type I: Long-duration smooth decrease [Figure 4].

Type II: Long-duration decrease with sporadic peaks [Figure 5].

Type III: Decrease with multiple peaks [Figure 6].

Type IV: Increase with multiple peaks [Figure 7].

Type V: A combination of declining and rising EDA [Figure 8].

Below, we describe each type with a representative profile and use the interview data to explain the morphologies. All interview segments are reproduced as they were without grammar edits to preserve authenticity. The rest of the profiles for all categories can be found in the Supplementary Material, Section SI.

#### 4.2.1. Type I: Long-Duration Smooth Decrease

In such a time series, we observed a long-duration decline in EDA values, indicating sustained disengagement with the topic. The qualitative data helped us validate this premise and understand the nature of disengagement. The participant watched the third video extracted from the film “Interstellar”. The sustained disengagement was a surprise, as one might expect more engagement from a movie than from video lectures [Figure 4]. The participant reflected below when asked about the video segment from 99 s to 385 s.

Interviewer: “What has happened in this Video?”

P2: “This video was not giving much information, I was able to understand few scenes, because I have watched the movie, however, I don’t remember this part, because I have watched this movie long back, but within this video clip, I don’t think I was able to understand or relate to any concept. I was expecting a connection with the previous videos, But I didn’t get any connection with the clip”.

Therefore, the participant tried to relate the concepts in the movie to the academic content of the previous two videos. When they failed to make that connection, it created a sustained disengagement, reflected in the EDA. Notably, this behavior was outside our expectations, as we had included V3, a movie segment with prominent actors in them, to foster more engagement and not less, as observed. However, the interviews revealed the reason for this apparent anomaly.

#### 4.2.2. Type II: Long-Duration Decrease with Sporadic Peaks

This category reflects another prevalent type of EDA data, where there is a decline after an initial rise or not. This decline is marked by sporadic peaks in between [Figure 5]. Such data beg the question of whether those peaks are significant or what they might indicate in the broader context. The initial drop in EDA could be attributed to the participant feeling overwhelmed at the beginning of the video (105 to 137 s), as indicated by their statement.

Interviewer: “What were your thoughts at the beginning of the video?”

P6: “They were many things written on the slide, whenever he says something, I was looking into the board, but as soon as I go there, I miss whatever he was saying, then again I go into the slides, this slide was little overwhelming”.

Thereafter, we see the surge in EDA with the sporadic peaks. When asked about the duration of the video segment, from 150 s to 213 s, of the two peaks, the participant reflected as follows.

P1: “He said that there were few concepts in physics which are unanswered, so I was a little curious about what is unanswered, then I started listening to him more, because I felt like let me see what is unanswered” [first peak].

P1: “When he was explaining that electric and magnetic fields will actually increase and decrease the magnitude with respect to the position with respect to space, but they may not change with respect to time, I was listening to him and actually I was very curious and I wanted to understand the diagram on the slide because it looks like a DNA structure, he started explaining the diagram, I was very happy, I was trying to align whatever he was trying to say, this part was actually very good for me” [second peak].

The statement above implies that the curiosity evoked by the content drove the participant’s engagement. The participant reflected the following when asked about the video segment from 245 s to 425 s.

Interviewer: “What has happened during this video segment?”

P1: “first of all I felt that, he was using too much of text on slide and he just reading the content, I was just listening, because I was also looking at the slide at same time”.

Therefore, it is clear from the participant’s responses that they paid attention when the video sparked curiosity, as indicated by the significant peaks in EDA. However, the participant watched passively and seemed disinterested throughout the video. Hence, it is crucial to recognize that while sporadic spikes in the EDA data indicate brief moments of engagement, these instances may not lead to sustained or meaningful engagement over time.

#### 4.2.3. Type III: Decrease with Multiple Peaks

The data could contain multiple peaks instead of sporadic peaks with decreasing EDA values (Figure 6). As peaks represent engagement, the overall decline despite so many peaks could pose a conundrum and require further inquiry.

When asked about the video segment from 120 s to 155 s, the participant reflected upon the following:

P7: “Schwarzschild radius is a new concept and new term for me. I was excited for whatever was coming”.

When asked about the video segment from 160 s to 560 s, the participant reflected:

P7: “Not that interesting, gravitational lensing and the photon sphere. It was specifically about light. So, I was like, not that interested”.

Interviewer: “Do you like the animations used in this video segment?”

P7: “I don’t love them, it’s okay for reference”.

Interviewer: “Were you judging the quality of those animations?”

P7: “Yes (smiling) I was judging those animations, they could have been better”.

Interviewer: “Here he is explaining a theory you already knew. So how did you feel?”

P7 “I was still enjoying it. It was to refine the whole thing. I don’t actively think about it and at dumb hole topic I lost my interest”.

The participant seemed excited about several things mentioned in the video, but the dialogue also suggests they were not as engaged with the topic. No indications of sustained engagement were found when prompted to consider other video portions. The presence of numerous animations within the video may have sparked moments of interest. This may have been the possible reason for the multiple peaks in EDA, but such engagement was again momentary and failed to sustain a meaningful connection with the content. Overall, the trend consistently showed a downward trajectory throughout the video. Notably, the survey data failed to capture the nuance brought out by the interviews. P7 classified this video as both cognitively and affectively engaging, with a score of 7 out of 7 for each. Yet, the interview segments suggest a much lower engagement level, which reflects the EDA characteristics much better. 

Therefore, the survey and the interview capture different facets of the candidate’s engagement profile. When asked in a survey, P7 could have recalled the connection to individual elements of the video. Still, due to a lack of fundamental interest in the topic, he revealed his general lack of interest through the interview data. Therefore, in terms of engagement, this would score over any curve showing a smooth decline (Type 1) or few peaks (Type 2). This was also represented in the mean EDA calculations, although no statistically significant difference could be ascertained: Type I M = −0.420, SD = 0.076, Type II M = −0.20, SD = 0.34, Type III M = −0.12, SD = 0.10, Type IV M = 0.11, SD = 0.19.

#### 4.2.4. Type IV: Increase with Multiple Peaks

Contrasting the previous three categories, this morphology shows an upslope with multiple peaks (Figure 7). As upslopes and peaks in EDA data should signify engagement periods, such data could indicate the maximum engagement possible for a participant and something to look forward to in educational or related media. The video was of the lecture, and the participant was a student of physics who, unlike the other participants, had taken courses on relativity in college.

When asked about the video segment from 97 s to 430 s, the participant reflected upon the following:

P5: “All the time I was kind of thinking and going back to the prior knowledge that I have about physics. And these experiments, also, I’ve read like Michelson Morley experiment and Maxwell’s theory of electromagnetism. I’m aware, so I was just kind of going back. I was trying to connect, like what I remembered, and I remembered a large chunk of it”.

When asked to rate the video in terms of cognitive and affective engagement, P5 rated it as 7 and 2 out of 7, respectively.

#### 4.2.5. Type V: A Combination of Declining and Rising Electrodermal Activity (EDA)

We have also noted a combination of the above categories. Most significantly, there were some profiles where an initial disengagement showed substantial recovery toward the end [Figure 8].

For example, P4 noted that they were initially (125–430 s) averse to listening deeply because they were distracted and/or found the concepts too difficult.

Interviewer: “What was your experience during this segment of the video?”

P4: “In this part I was distracted and felt monotonous, unable to understand anything, tried to understand but could not”.

Interviewer: “Were you able to understand the concepts discussed in this part of the video?”

P4: “No, I was just looking at the animations”.

The statement above indicates that the participant experienced distraction and monotony during this segment of the video, indicating a lack of engagement with the content. They expressed difficulty in understanding the concepts discussed and focused solely on the visual animations.

However, in this video, the topic of wormholes comes up at 545, after which P4 clearly recalled feeling engaged.

P4: “After seeing wormhole topic, I thought, I should try it again and let’s focus again”.

Interviewer: “Were you interested in wormholes?”

P4: “You can say that, before mentioning of wormhole topic, I did not paid any attention, it was like trigger point, I felt that, I should not lose the attention, lets focus”.

The statements imply that the participant consciously tried to reorient their focus and engage with the content upon encountering a specific topic. This proactive decision suggests a deliberate attempt to reignite their attention and delve into the presented content. Subsequently, they demonstrated a brief but notable period of engagement with the video, indicating a responsive interaction with the content, as evident in the surge in EDA around 550 s.

### 4.3. Latency Estimation

The latency period is a measure of the time between the onset of a stimulus and the initial discernible change in the electrodermal signal. This period captures the delay in the physiological response of the skin’s sweat glands to external or internal stimuli [57,58,59]. This duration depends on the individual and the presented stimulus, with the other factors of electrodermal activity being variable and influenced by the person and the situation [58,59]. The latency of electrodermal activity (EDA) is typically between 1 and 5 s after the onset of a stimulus [57,59].

Our study provided a unique opportunity to measure latency. For most videos, the transition from baseline to stimulus shows a marked rise (for example, in Figure 5, Figure 7 and Figure 8 above). The transition from gray screen to video is both stark and precise. Hence, we could establish point estimates of the lag time. The mean latency for video 1 was observed to be 2.62 s, 1.96 s for video 2, and 4.04 s for video 3. The overall mean latency was 2.87 s, with a standard deviation of 0.86.

## 5. Discussion

In this study, we aimed to understand Electrodermal Activity (EDA) using a mixed-methods research design focusing first on collecting and analyzing quantitative data, followed by collecting qualitative data to deepen the interpretation of the findings.

We quantified the mean Electrodermal Activity (EDA) and observed a correlation (marginally insignificant despite the low sample size) with cognitive engagement (r(19) = 0.416, *p* = 0.061). However, our analysis revealed no noteworthy correlation between mean EDA and affective engagement (r(19) = 0.0197, *p* = 0.933). These findings align with the existing literature, highlighting the effectiveness of EDA as a measure of cognitive engagement but not affective engagement [16,60,61,62]. The average latency observed in this study was 2.87 s, which aligns with the range reported in the existing literature on EDA’s latency [57,59].

Those metrics provided key insights and validated past work on EDA. However, EDA is still poorly understood and consequently not used widely. The causes behind the short-term variations and long-term trends remain unexplored. This work was primarily geared to speak to this gap. We used a mixed-methods research design to probe into the quantitative EDA data with qualitative interviews, aided suitably by additional survey data and questionnaires.

Twenty-one EDA profiles from participants watching three videos were generated. These profiles could be categorized into five broad morphologies based on the EDA and interviews, where each morphology represented a unique manifestation of engagement-disengagement dynamics. In Type I (n = 3), sustained disengagement was found to be reflected through a long-duration smooth decrease in EDA. The qualitative data helped establish a lack of engagement, potentially indicating that the video failed to capture the viewer’s attention in any meaningful way. In Type II (n = 5), the profiles showed few peaks amidst an overall decrease. Such long-duration decreases with sporadic peaks indicate a subtly different engagement profile. The interviews revealed somewhat more engagement than Type 1, but the cause of engagement was not necessarily about the video as a whole. Instead, the peaks reflected isolated arousal points due to graphics, animations, explanations, or noise. However, such moments of heightened physiological arousal amidst an overall decline in EDA suggest that the intermittent short periods of engagement could not be sustained to create broader engagement with the content. Type III (n = 3) demonstrated a downward trajectory with more peaks than Type II. Such a decrease with multiple peaks in EDA signified more engagement, and the interview data corroborated this assumption. The peaks coincided with emotionally impactful content or instances of active cognitive processing. Type IV (n = 4) differed clearly from the other three in that they represented the only cases in the dataset where the data showed a positive slope from an increase in EDA along with multiple peaks. The interviews revealed strong and sustained engagement, and survey data also indicated deeper cognitive processing. Type V (n = 6) represented a combination of these profiles, primarily the same profile showing initial disengagement followed by engagement or vice versa. Notably, most EDA showed a dominantly disengaged profile. We can extrapolate our findings to suggest that engagement profiles also show familiar variations when sampled with proper video–participant pairings.

The Type 1 and Type 4 examples make for an exciting case of contrasts and again corroborate, as mentioned earlier, what is stated in the literature—EDA is a measure of cognitive engagement but not affective engagement [16,60,61,62]. While we expected more engagement than what is observed in P2-V3 (Figure 4) as it was from a movie, the interview made it clear that the student was not cognitively engaged. For P5-V1 (Figure 7), we observed the reverse scenario, where a complex and apparently less affectively engaging (compared to a movie segment) video produced engagement because P5 was cognitively engaged but not affectively, as observed in the survey and interview data (Section 4.2.4).

The detailed mixed-method analysis reveals the critical balance of short-term arousal signatures and long-term trends in EDA data. We need to keep this fundamental insight in mind to predict engagement–disengagement in such contexts. The short-term spikes may provide isolated arousal from superficial engagement with the content, but the longer wavelengths determine the actual engagement. That is not to say, however, that these peaks do not indicate meaningful engagement at all, as they do play a significant role (for example, in Type III and IV). Therefore, for us to be able to predict when a student will be engaged/disengaged, we need to find a metric that charts the evolution of these two types of variations. Such a metric would allow for real-time monitoring of engagement levels, enhancing the customization of learning experiences and interventions, such as that required for a truly adaptive Intelligent Tutoring System (ITS).

We propose a contextually novel metric—the moving average crossover—to capture long-term trends amidst short-term, less meaningful peaks. This metric can more effectively predict participant engagement or disengagement by comparing two averages computed over different timeframes. This is a common technique in analyzing financial data trends [63]. In financial data, when the shorter time period average crosses the long-term average from below (known as the golden crossover), it indicates a potential reversal of the trend from bearish to bullish. In contrast, the opposite scenario (the death crossover) suggests a shift to a bearish trend. The moving average crossover offers valuable insights into potential trend reversals based on the historical price movements of securities [64]. We also find the application of crossover moving averages in supply chain management [65] and health care [66].

To illustrate the concept in the current context, we use moving averages of two time frames—30 s and 3 min. The time periods have been chosen arbitrarily to demonstrate the concept. In our context, corresponding to the golden crossover in financial analysis, when the shorter-term average rises above the long-term average, we get an engagement crossover. Conversely, when the long-term average exceeds the shorter-term average, it is termed a disengagement crossover.

In all types of profiles, we see the crossover capturing major engagement–disengagement periods within a reasonable lag post-onset. In Type I, we have the least issue with false positive signals; a sustained profile of this kind would be the easiest to monitor (Figure 4). In Type 2, the sporadic peak may pierce through the SMAs and thereby create some false positive signals; however, the chances are still limited (Figure 5). Type 3 and 4 are most susceptible to false positive signals. The prediction would be most beneficial for Type 5 profiles, as the crossover should be able to predict long-duration changes with a fair amount of accuracy and minimal false positives (see Table 2 for a comparison). As this metric can be calculated without baseline measurement, it is easy to operationalize and reduce data collection overheads. The calculation of moving average crossovers utilized two averages derived from the same dataset. Hence, this method ensured that the crossovers occurred at the same points on the *x*-axis, regardless of baseline correction, which affects only the ordinate of a time series.

## 6. Conclusions

In this study, we used three related stimuli and a group of seven participants to understand commonly occurring EDA morphologies. We could categorize all EDA profiles into five major types, which showed the role of both short-term and long-term dynamics in contributing to the total EDA. This understanding laid the groundwork for suggesting the simple moving average crossover as a predictive metric for engagement or disengagement. Our study adds to the existing literature in three ways.

First, we use qualitative data to better understand subtleties in EDA signal variations. While similar approaches have been explored in earlier studies [53,54], we extend these methods by introducing new constructs and providing a more extensive dataset for analysis.

Second, we present 21 meticulously annotated EDA profiles in the Supplementary Document. This repository is intended to serve as a resource for future researchers interested in investigating EDA data related to engagement. Although our database is limited and the five categories are not exhaustive, as acknowledged in our limitations, it represents a foundational starting point for further exploration.

Lastly, our proposal to utilize moving average crossovers for EDA analysis in measuring engagement represents a significant innovation. Traditional EDA metrics, such as the average EDA, number of peaks, SCL, and SCR, are currently being explored for integration into Intelligent Tutoring Systems (ITS) to gauge cognitive load, engagement, and arousal. However, these metrics are poor in detecting changes in the signal. The crossover technique detects changes in trends by acknowledging short-term fluctuations and long-term variations. This approach could offer a more effective alternative to traditional metrics, for which benchmarks are hard to obtain. Crossover utilizes short vs. long-term variation in a dataset of the same individual, thereby circumventing more complex assumptions and benchmarking. Notably, the calculation of the moving average crossover did not rely on baseline data, and the novel metric effectively predicted the engagement and disengagement periods across all types of stimuli.

## 7. Limitations and Future Recommendations

EDA data suffers from artifacts resulting from participant activity. Despite controlled conditions such as instructing participants to refrain from moving their hands with the EDA sensor, placing the sensor on the non-dominant hand, and conducting the experiment in a temperature-controlled room, complete noise exclusion is hard to achieve. While these factors might have influenced our results, their impact was negligible since such noise artifacts would not have appeared prominently enough to merit the qualitative exploration in the study.

Another significant limitation of this study is the small sample size in terms of participants and video types. Although we have successfully established quite a few morphologies credibly, we are certain that this list is not exhaustive. Therefore, future studies should include larger sample sizes and diverse contexts to validate and extend the findings on EDA morphologies and engagement profiles. More importantly, most of the profiles were dominantly disengaged, and although we surmised that engagement profiles would behave very similarly, we cannot be certain with this limited sampling. Also, such a small dataset can only partially rule out participant bias. For example, the large majority of data shows disengagement of some kind, thereby limiting our findings. For future studies, we advise a more extensive study concerning both the participants and the stimulus type so that the participant–stimulus space is varied enough to elicit all kinds of engagement. Also, we have structured our design to elicit arousal through engagement primarily. This approach could be potentially expanded to include other constructs like cognitive load, stress, etc.

We have suggested the use of moving average crossovers in this study. However, we have not established the most suitable window size for its calculation. Neither have we provided prediction statistics, such as precision, accuracy, or F1 values. Future studies would need to conduct more statistically robust estimates for these parameters. Furthermore, other types of moving averages, such as exponential, could be explored in the same context.

## Figures and Tables

**Table 1:** Representation of the codebook utilized in the study.

Code	Sub-Code	Definition	Example from Transcript
Disengagement	Disconnection	Represents instances where the participant mentally or emotionally withdraws from active engagement in the interaction or topic under discussion.	But I can’t say that I was truly connected. I could not connect much. I can see that this thing’s what he’s saying. I just remember
	Aversion	Indicates a withdrawal from active participation in the current interaction or task.	I have watched the movie, but there was some confusion in my mind. But I thought this clip is from inception movie.
	Passive Watching	This signifies casually observing without actively participating or engaging.	I was curious to understand how will they implement the plan to reach the miller planet, there was a suspense what will happen next?
Engagement	Curiosity	Represents active engagement driven by genuine interest or curiosity in the topic or conversation.	I was curious to understand how will they implement the plan to reach the miller planet, there was a suspense what will happen next?
	Recall	Represents when the participant tries to remember something from their prior knowledge	So, I remember, like, I don’t remember the specifics, but I‘ve heard of these things, so it was like I was trying to recall. Recall, yeah!
	Attention	Indicates when participants actively process information from the stimuli being presented	I noticed that. I paid attention. I noticed that it was an important thing to pay attention
Media	Sound/Video	When stimuli have created a sound which is higher level for the participant	Sound was loud, I think!

**Table 2:** Summary of moving average crossover prediction. Only those signals that indicate sustained (longer time frames) engagements are listed. The rest are either categorized under false positives (if EDA reverses direction within a few seconds) or discounted (if the direction is correct yet short-lived); for example, in Type II (Figure 5), sustained disengagement starts at 105 s and is predicted at 135 s. However, crossovers also occur at 157 s, 175 s, and 215 s. Only the one at 175 is listed as a false positive, and the other two are discounted.

Serial No.	Type	Disengagement Starts at (s)	Disengagement Predicted at (s)	Engagement Starts at (s)	Engagement Predicted at (s)	False Positive (s)
1	I	96	126	-	-	-
2	II			140	157	175
3	III	Multiple predictions, several false positives				
4	IV	-	-	95	125	135, 250
5	V			100	240, 260	155

## Data Availability

The original contributions presented in the study are included in the article/Supplementary Material; further inquiries can be directed to the corresponding author/s.

## Supplementary Materials

The following supporting information can be downloaded at: https://www.mdpi.com/article/10.3390/s24144565/s1, Section SI: Graphical Representation; Section SII: Qualitative Data; Section SIII: Reference Survey Form.
