# Supplementary material for: Electrodermal Activity (EDA) Morphologies and Prediction of Engagement with Simple Moving Average Crossover: A Mixed-Method Study
Source: Sensors (Basel). 2024 Jul 14;24(14):4565. doi: 10.3390/s24144565 (PMC11280656; doi:10.3390/s24144565)
Supplement: Supplementary file 1 [file sensors-24-04565-s001.zip › sensors-3018683-supplementary.pdf]

SUPPLEMENTARY MATERIAL

1. Introduction

The supplementary sections accompanying this document provide detailed qualitative data to complement the graphical representations. These guidelines are designed to facilitate seamless navigation and understanding of the relationship between the data in both sections I & II.

2. Section I : Graphical Representation

Section I consists of 21 graphs illustrating various aspects of the data. Each graph is labeled with a unique identifier such as "P1V1," denoting the participant (P#) and video (V#) associated with it.

To understand the qualitative data corresponding to a specific graph in Section I, refer to the label located in the top left corner of the graph and note the participant number (P#) and video number (V#) mentioned in the label.

For example, to understand the first figure in Section I, the first step is to refer to the label located in the top left corner, here it is P2V3. Now look for P2 under the Participant Column and V2 under Video Column in Section II. Now, as per the guidelines, look for the approximate time range; here it is 125 - 700 seconds. In this scenario, the interviewer has asked, 'What was happening in this video? Was it interesting for you?' and the participant briefly replied, 'I didn't pay attention. I don't think I got any content.' Therefore, it was coded as passive watching > disengagement and colored red in the figure.

Type I : Long duration smooth decrease

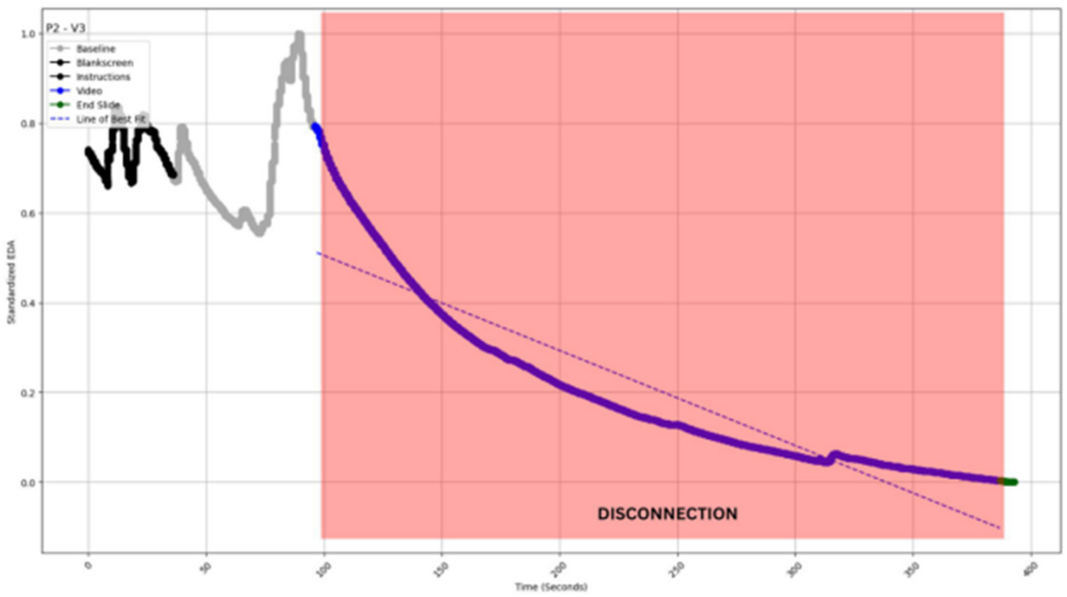

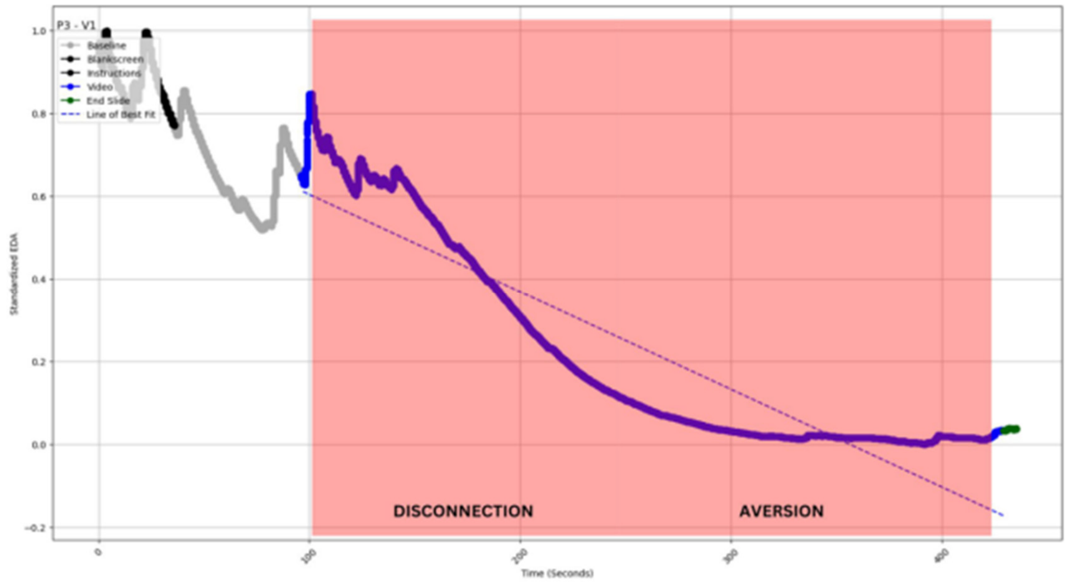

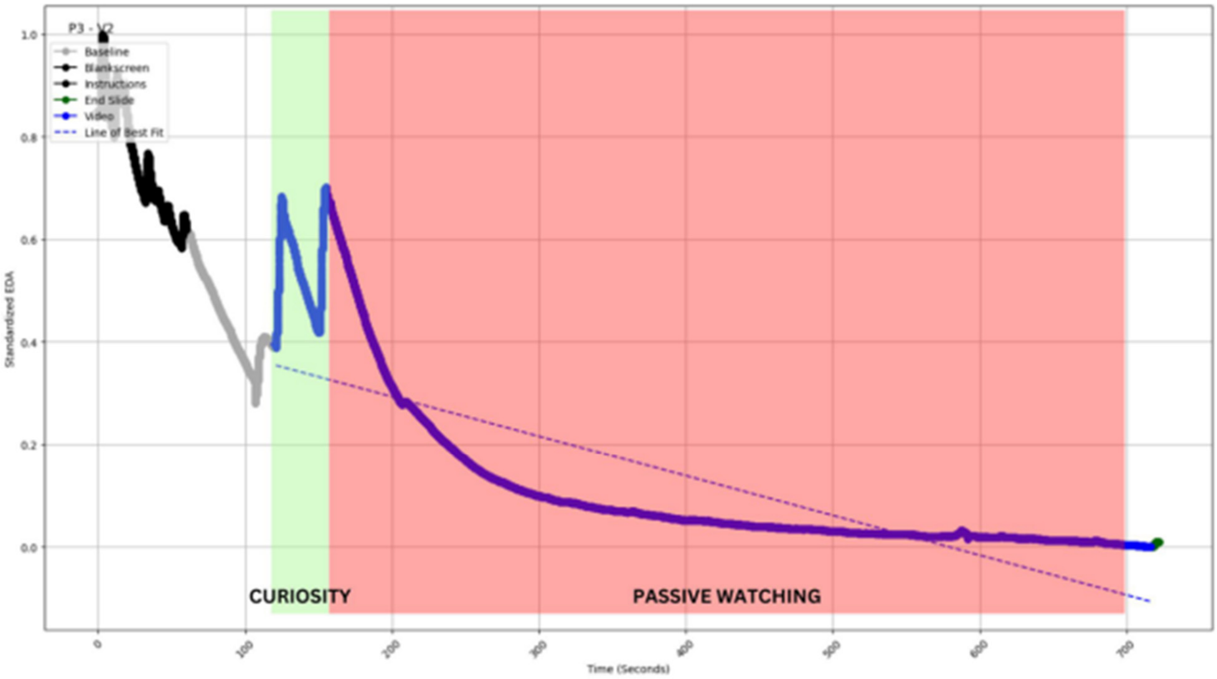

P3 - V2
Baseline
Blankscreen
Instructions
End Slide
Video
Line of Best Fit
Standardized EDA
Time (Seconds)
CURIOSITY
PASSIVE WATCHING

Type II Long duration decrease with sporadic peaks

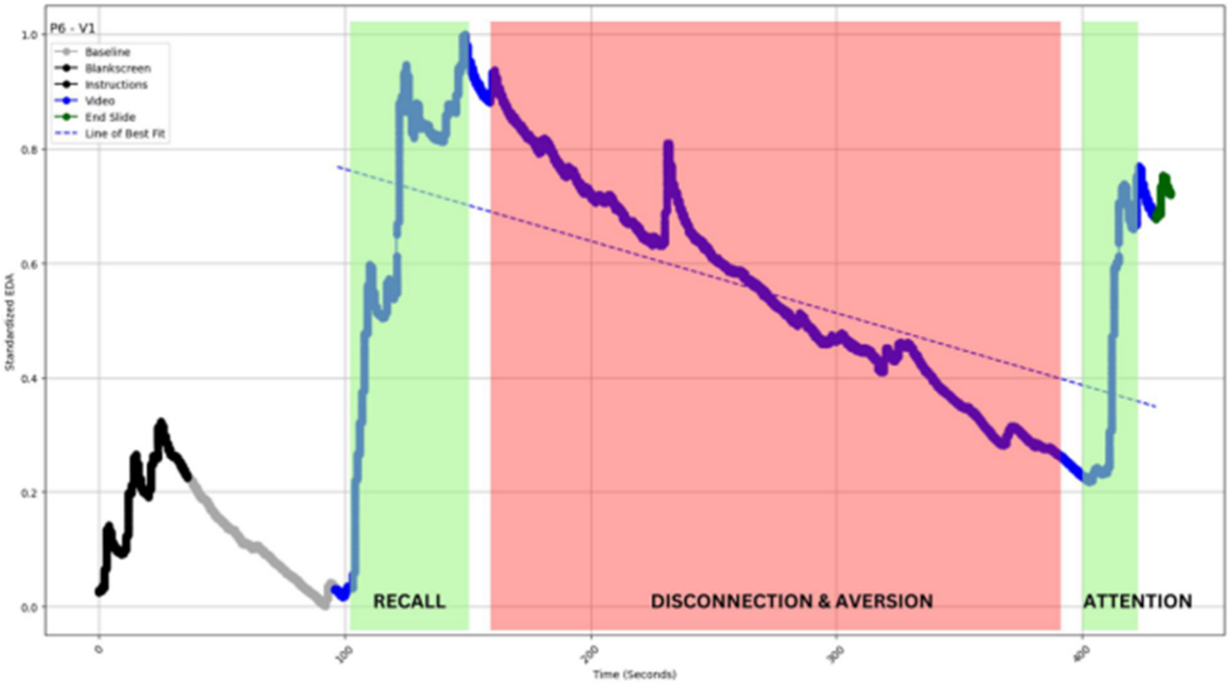

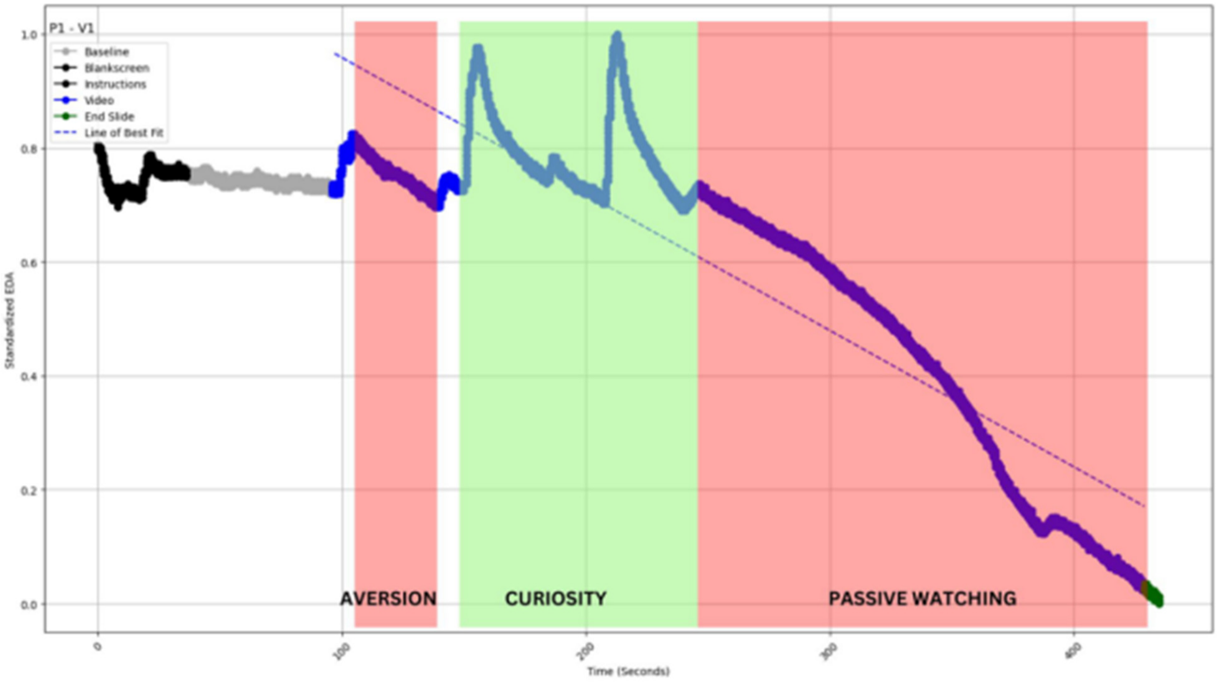

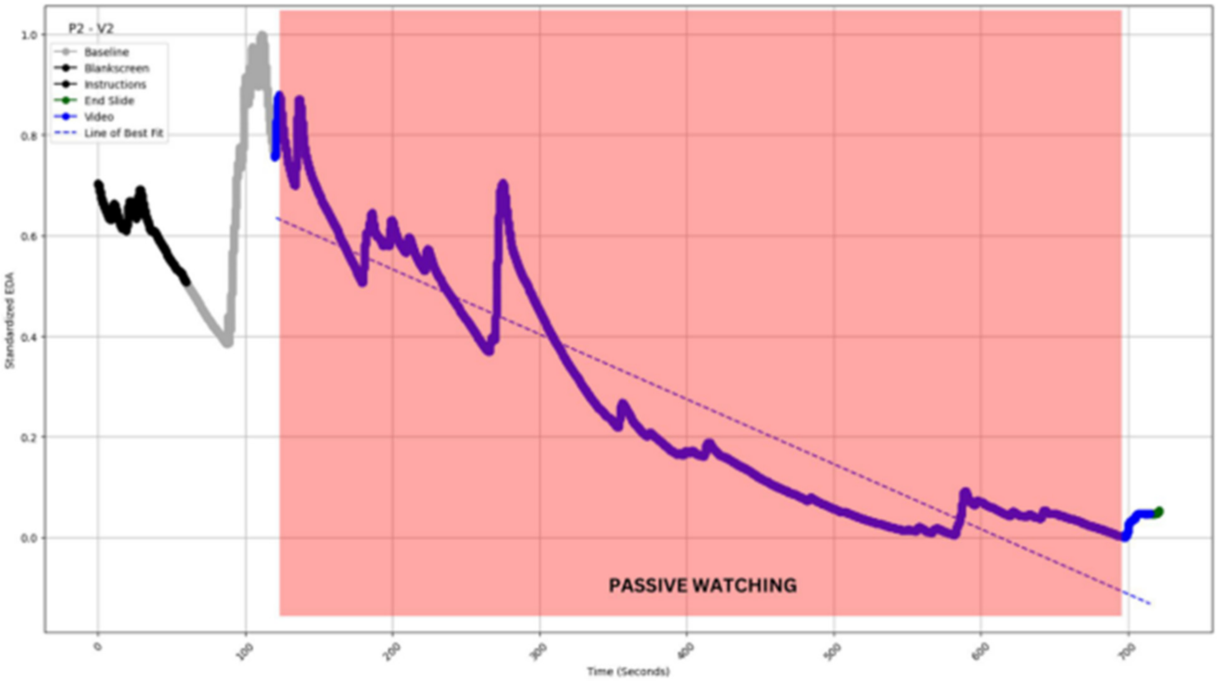
P2 - V2
Baseline
Blankscreen
Instructions
End Slide
Video
Line of Best Fit
PASSIVE WATCHING
Standardized EDA
Time (Seconds)

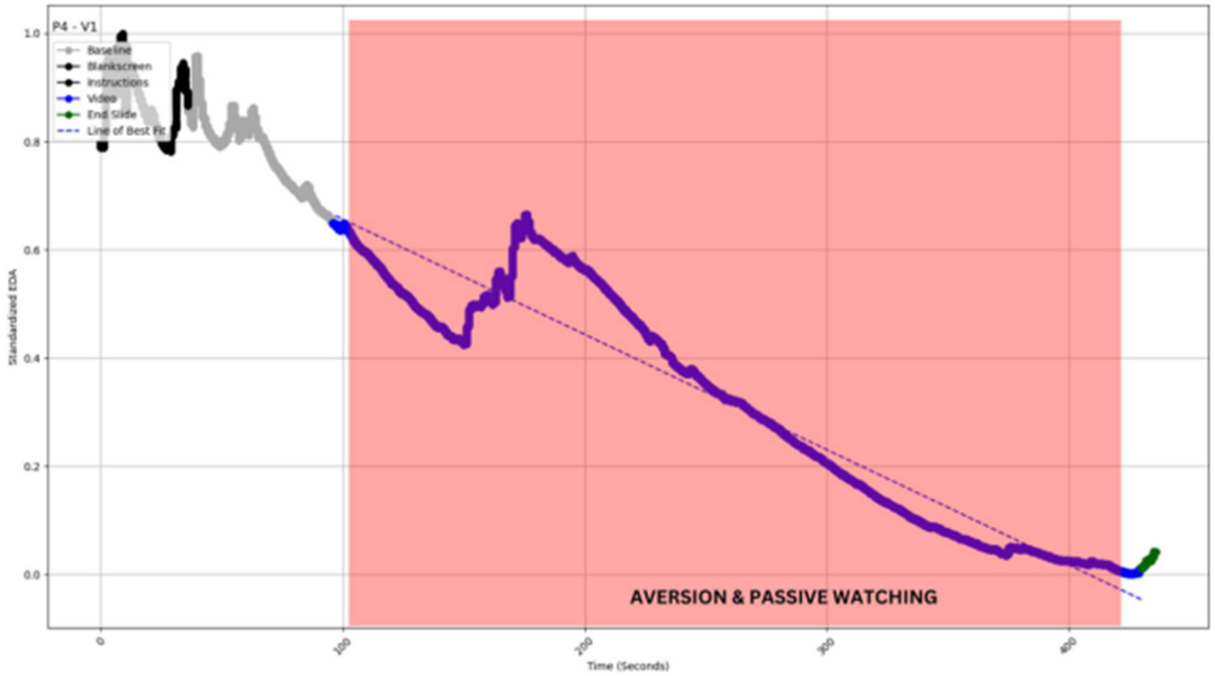
P4 - V1
Baseline
Blankscreen
Instructions
Video
End Slide
Line of Best Fit
AVERSION & PASSIVE WATCHING
Standardized EDA
Time (Seconds)

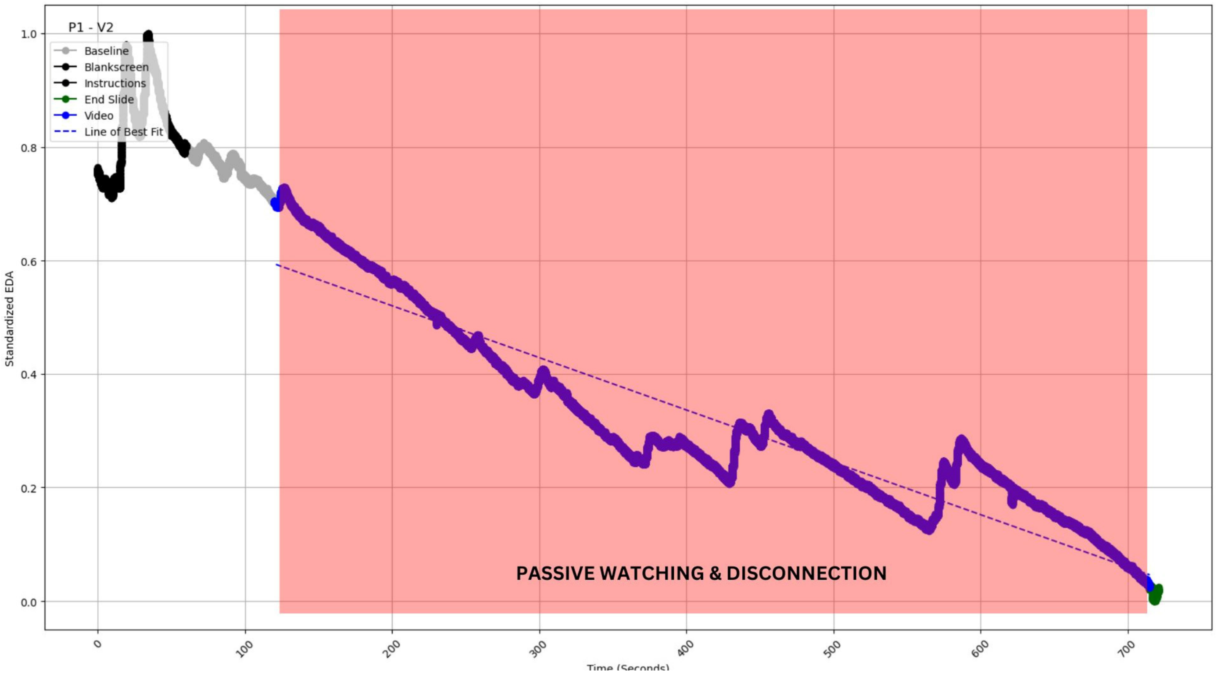
P1 - V2
Baseline
Blankscreen
Instructions
End Slide
Video
Line of Best Fit
Standardized EDA
1.0
0.8
0.6
0.4
0.2
0.0
PASSIVE WATCHING & DISCONNECTION
0
100
200
300
400
500
600
700

Type III Decrease with Multiple Peaks

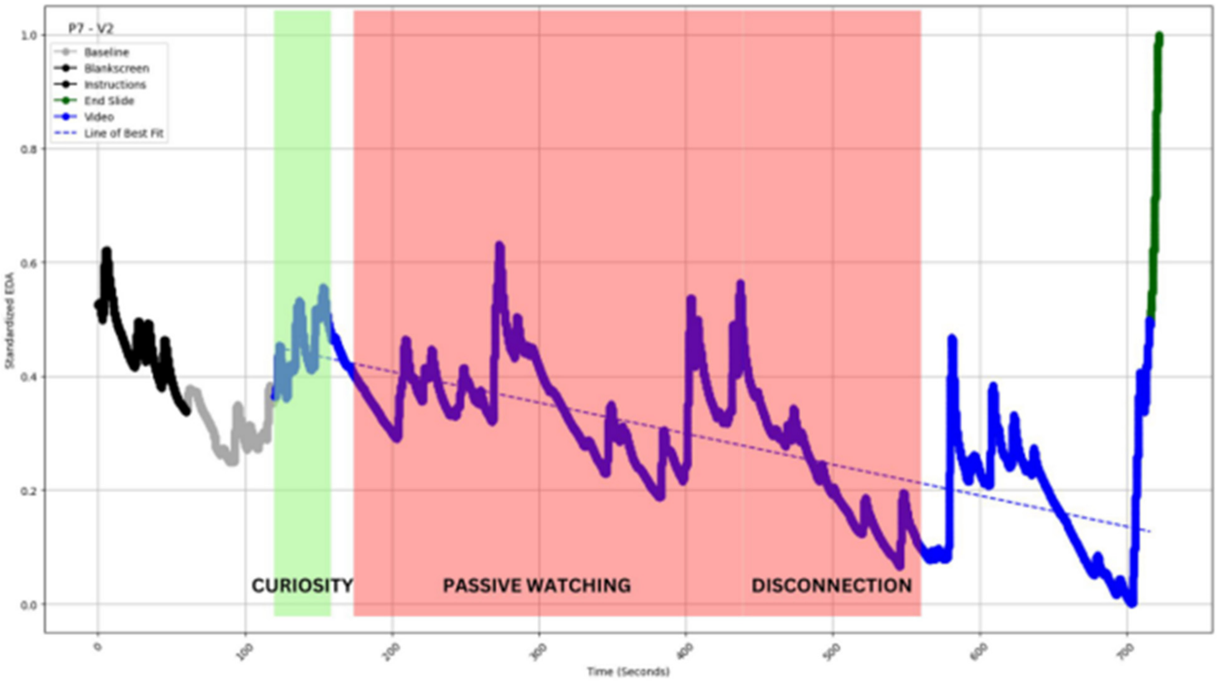

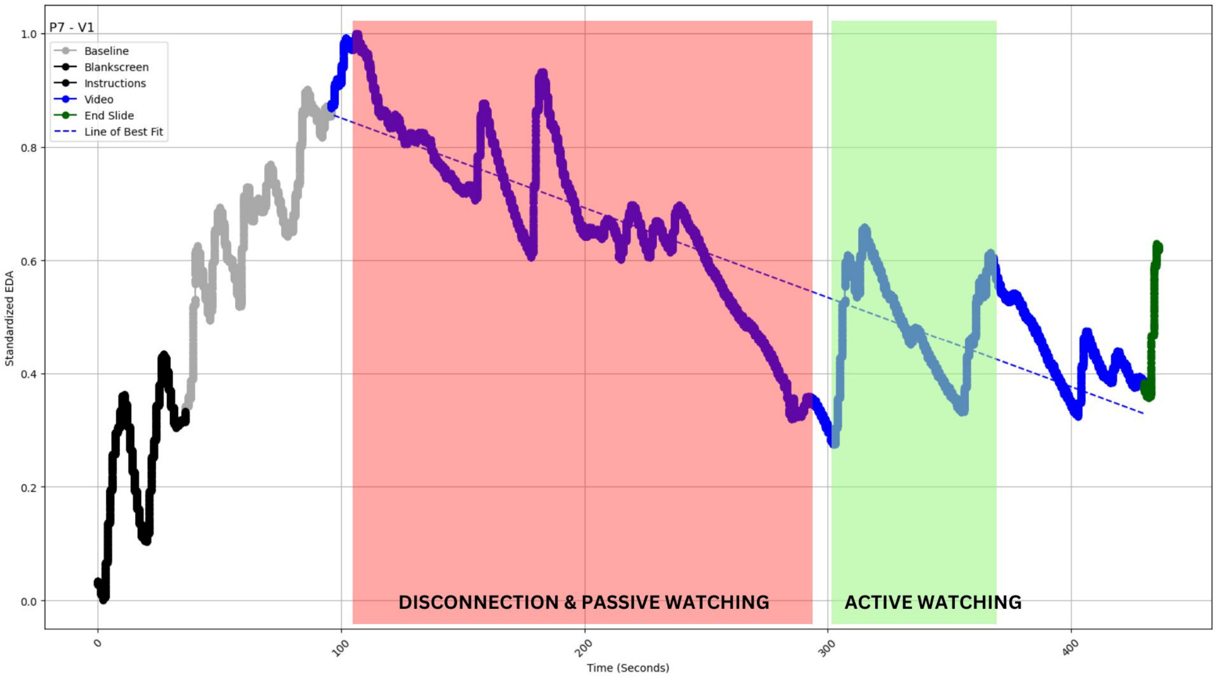
P7 - V1
Baseline
Blankscreen
Instructions
Video
End Slide
Line of Best Fit
DISCONNECTION & PASSIVE WATCHING
ACTIVE WATCHING
Standardized EDA
Time (Seconds)

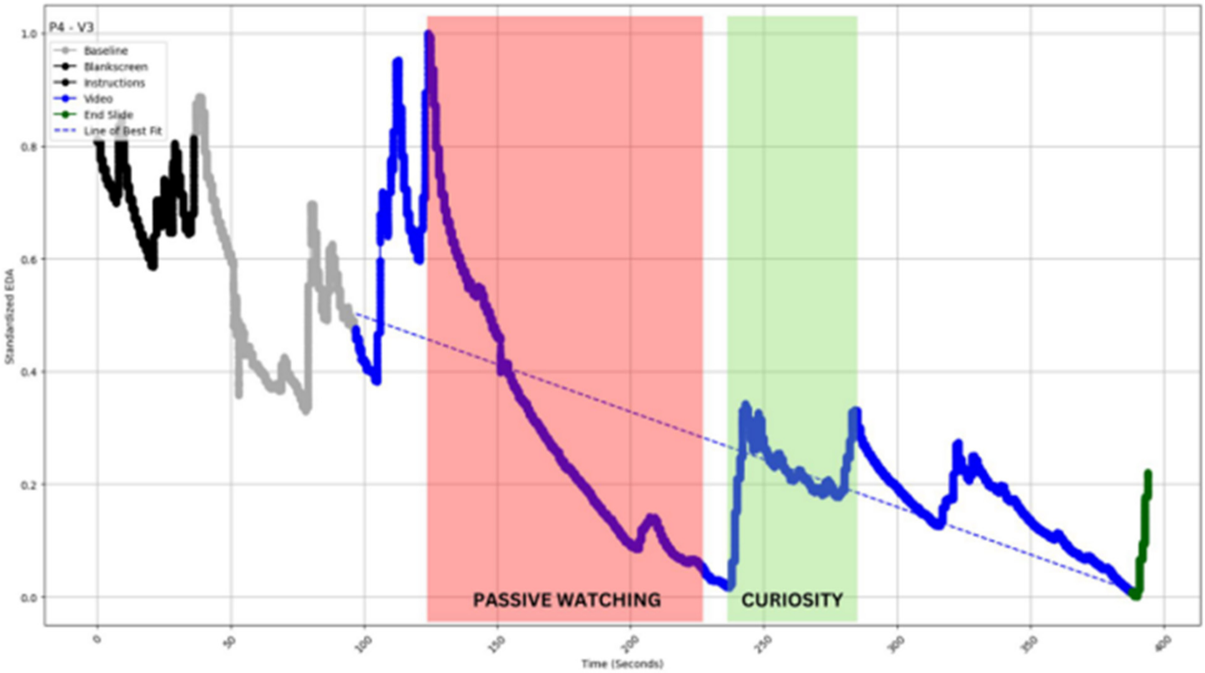
P4 - V3
PASSIVE WATCHING
CURIOSITY

Type IV Increase with multiple peaks

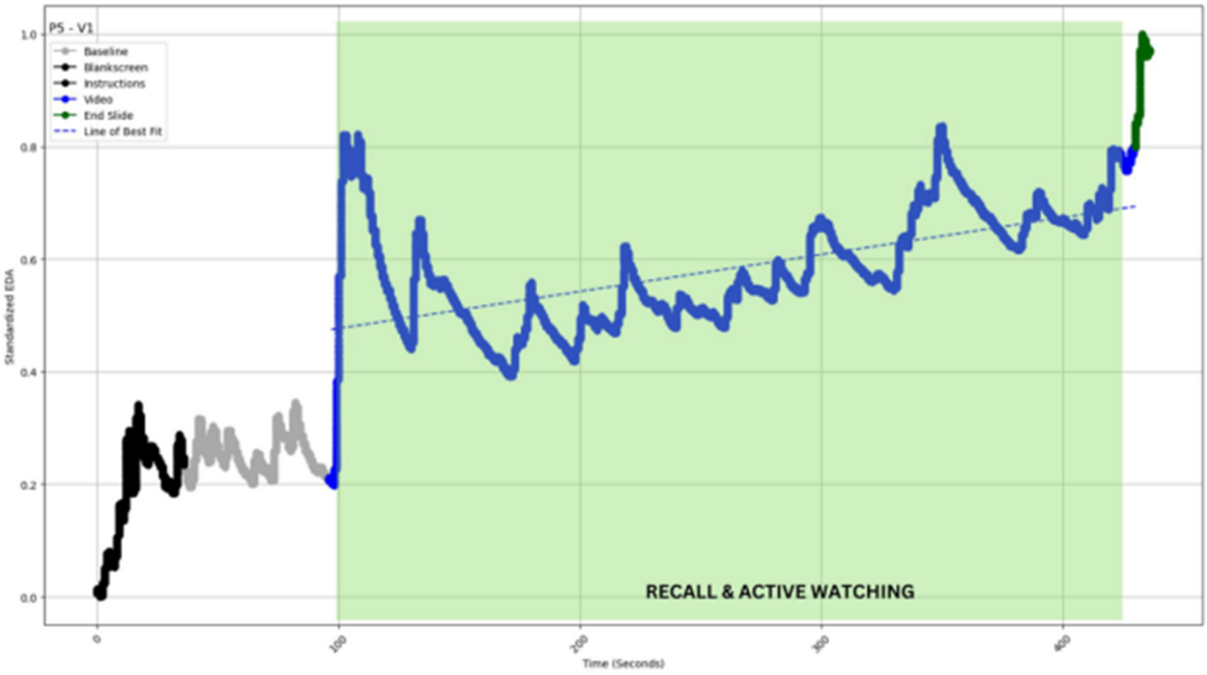

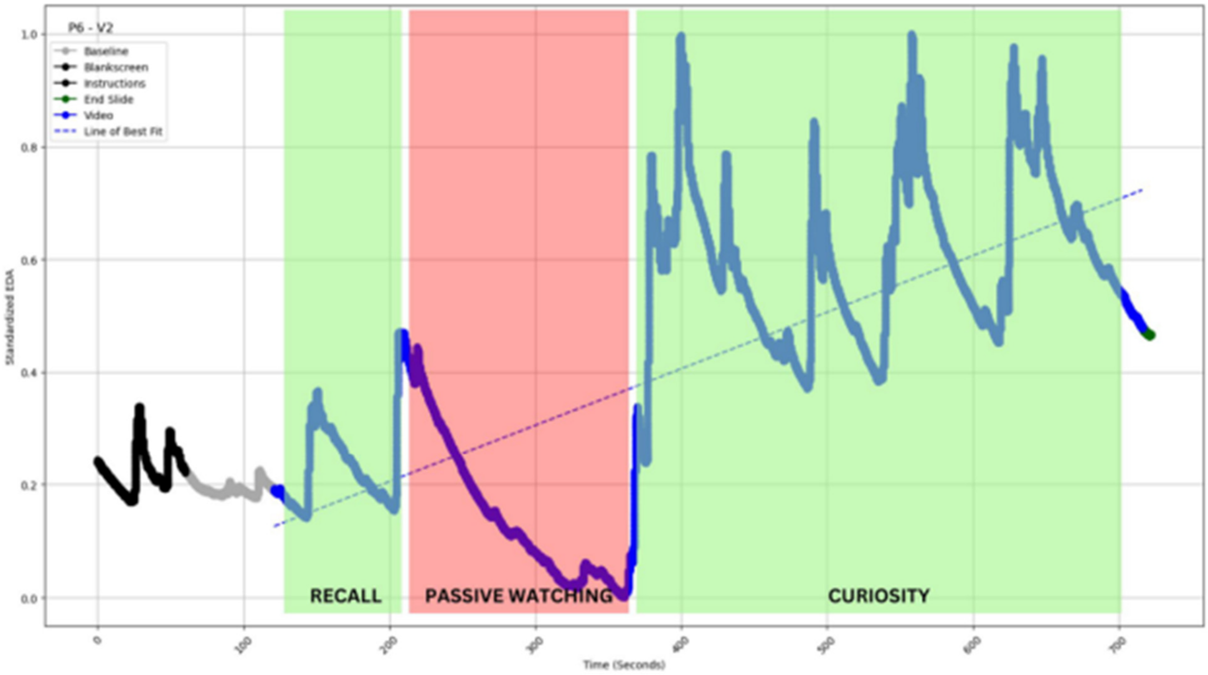

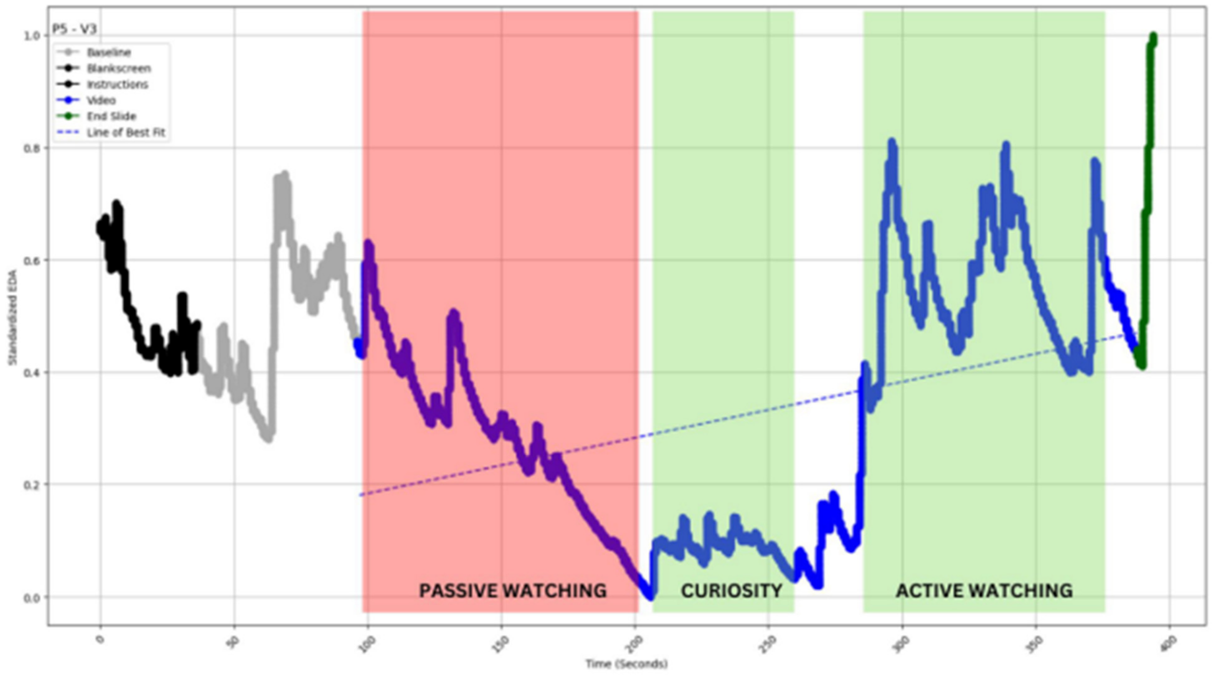
P5 - V3
Baseline
Blankscreen
Instructions
Video
End Slide
Line of Best Fit
Standardized EDA
Time (Seconds)
PASSIVE WATCHING
CURIOSITY
ACTIVE WATCHING

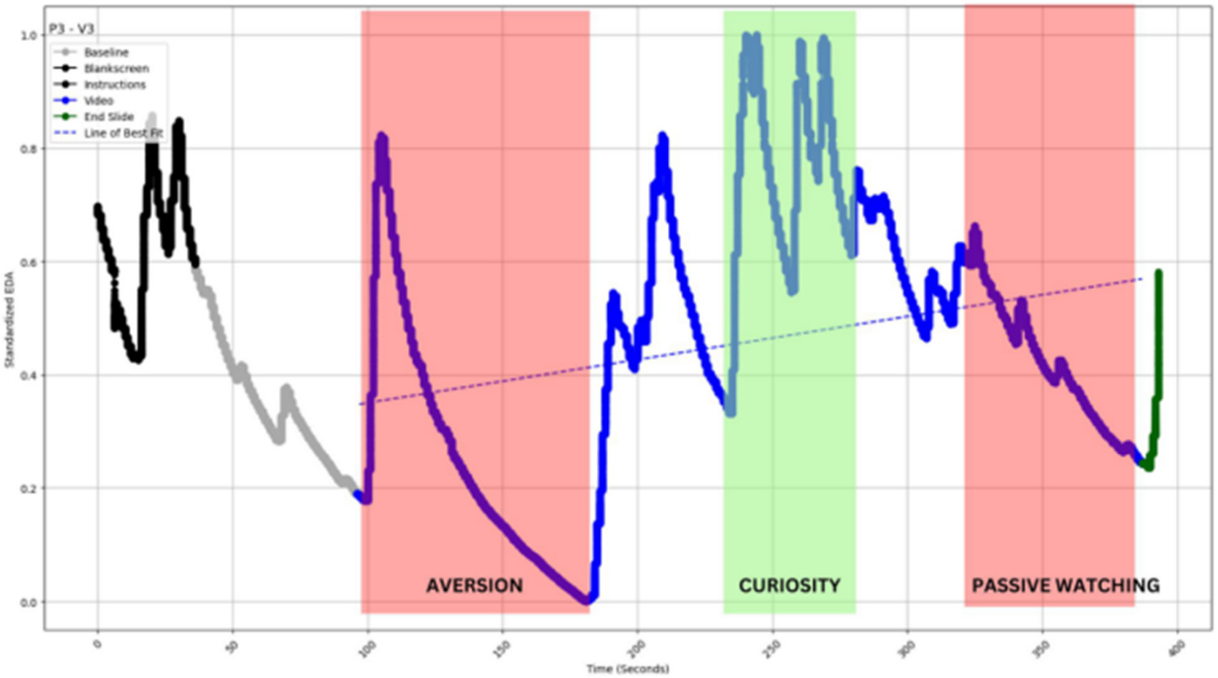
P3 - V3
Baseline
Blankscreen
Instructions
Video
End Slide
Line of Best Fit
Standardized EDA
Time (Seconds)
AVERSION
CURIOSITY
PASSIVE WATCHING

Type V: A combination of declining and rising Electrodermal Activity (EDA)

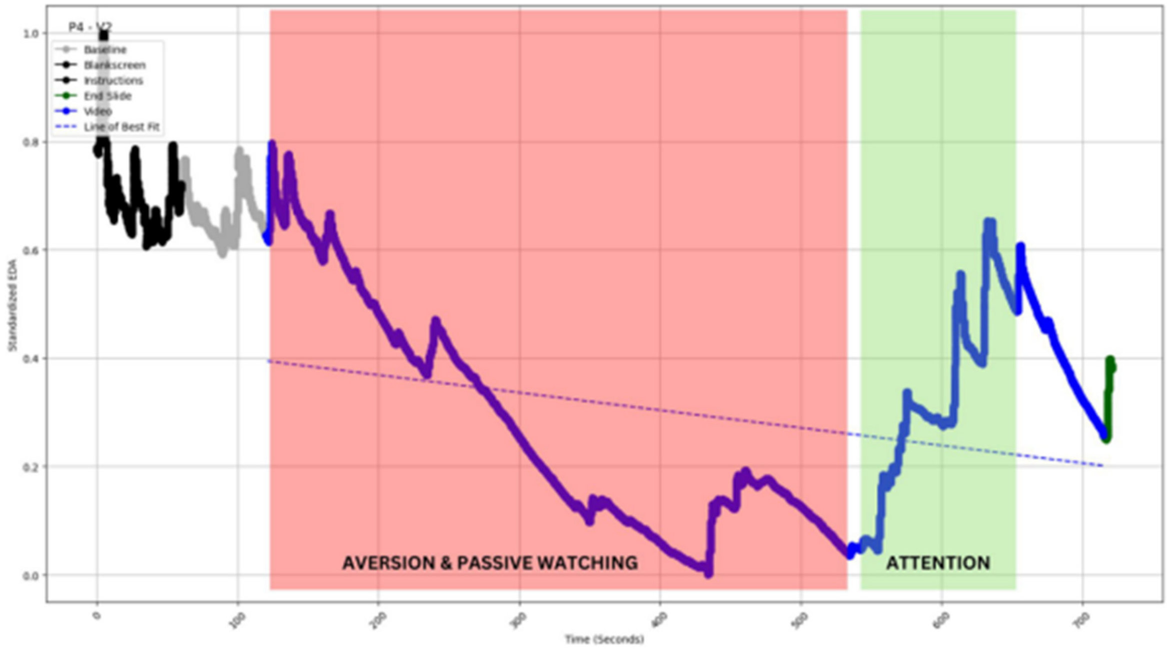

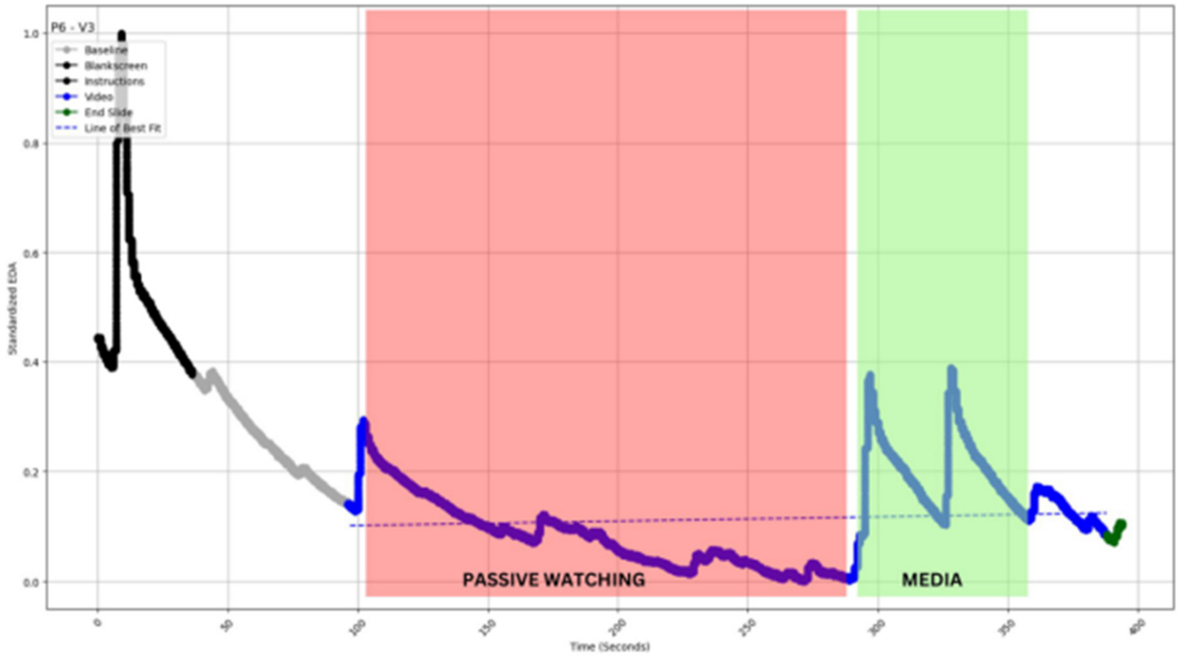

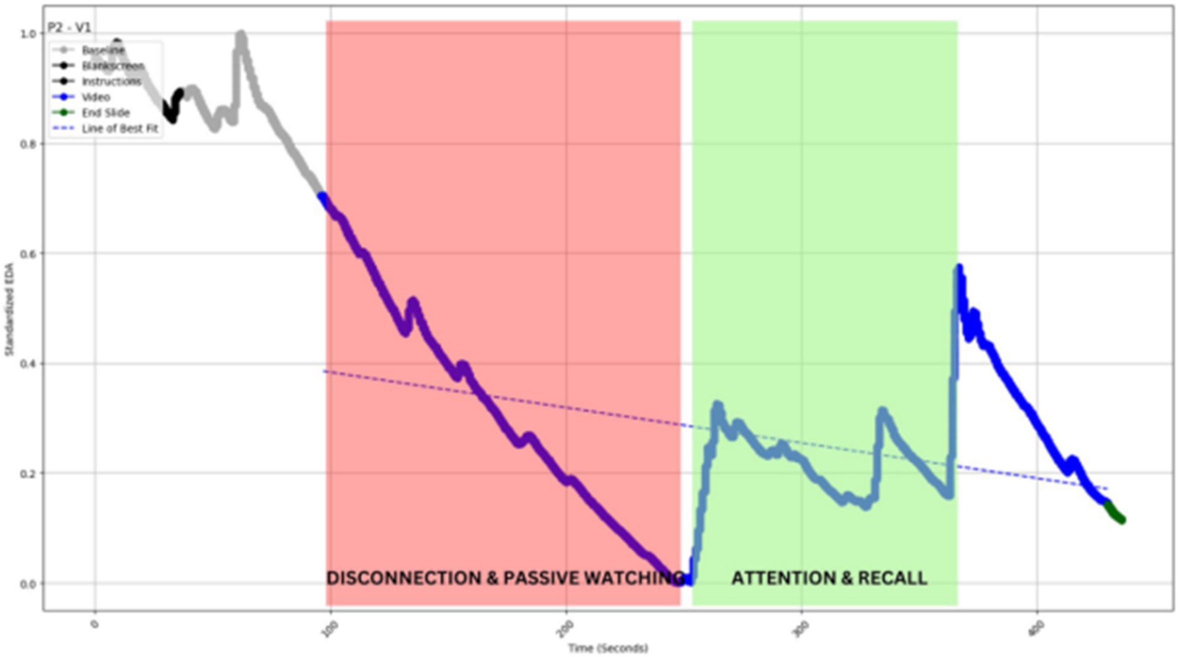
P2 - V1
Baseline
Blankscreen
Instructions
Video
End Slide
Line of Best Fit
Standardized EDA
Time (Seconds)
DISCONNECTION & PASSIVE WATCHING
ATTENTION & RECALL

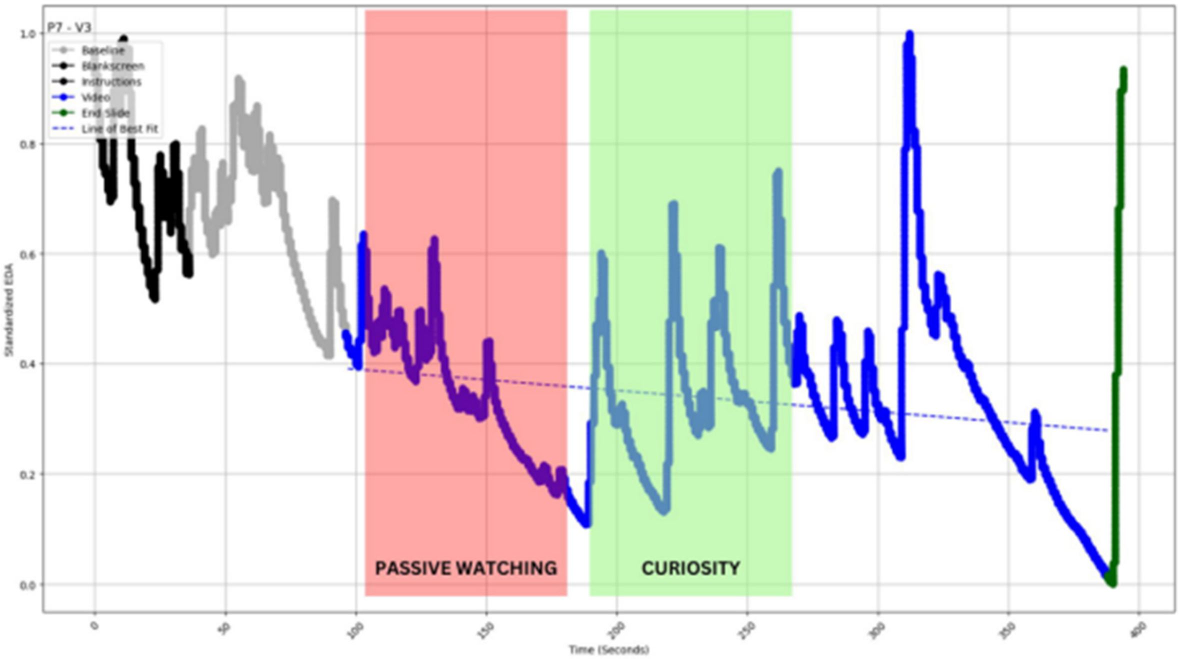
P7 - V3
Baseline
Blankscreen
Instructions
Video
End Slide
Line of Best Fit
Standardized EDA
Time (Seconds)
PASSIVE WATCHING
CURIOSITY

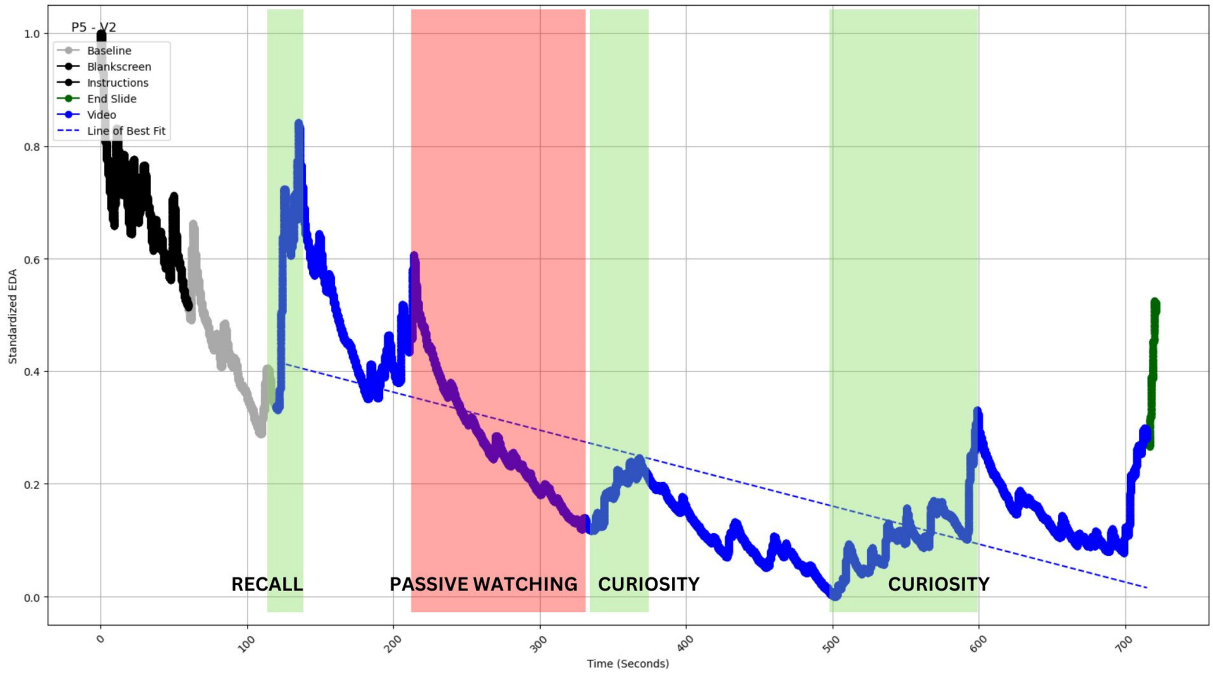
P5 - V2
Baseline
Blankscreen
Instructions
End Slide
Video
Line of Best Fit
Standardized EDA
RECALL
PASSIVE WATCHING
CURIOSITY
CURIOSITY
Time (Seconds)

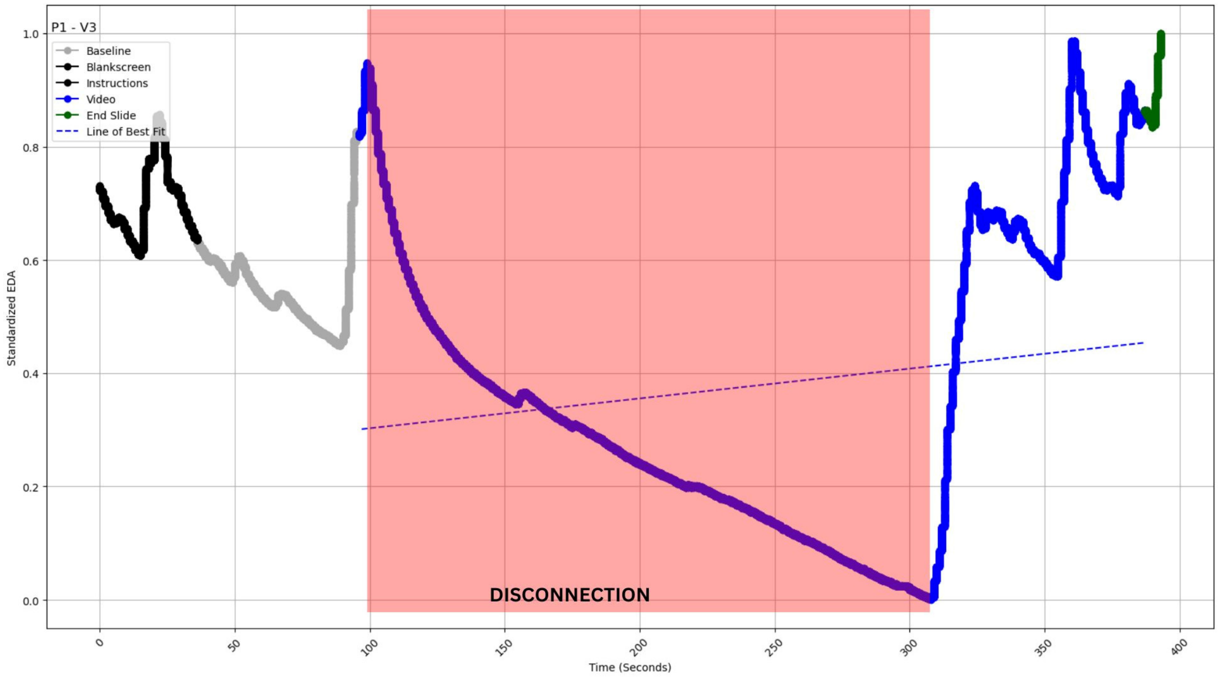
P1 - V3
Baseline
Blankscreen
Instructions
Video
End Slide
Line of Best Fit
Standardized EDA
DISCONNECTION
Time (Seconds)

Section II : Qualitative Data

Section II contains qualitative data corresponding to the graphs in Section I. This data is organized into columns including Participant, Video, Part, Time Range (in Secs), Interviewer, Participant Verbal, Sub-Code, and Code.

Comprehensive Codebook

Ser	Participant	Video	Time Range (in Secs) (Approx)	Interviewer	Participant Verbal	Sub -Code	Code
1	P1	V1	105 - 137	What were your thoughts during the beginning of the video?	They were many things written on the slide, when ever he says something, I was looking into the saying, but I soon as I go there, I miss whatever he was saying, then again I go into the slides,this slide was little overwhelming	Aversion	Disengagement
			150 - 213	Was it interesting ? or was it boring ?, what has happened during this video segment	He said that there were few concepts in physics which are unanswered, so I was a little curious about what is unanswered, then I started listening to him more, because i felt like let me see what is unanswered	Curiosity	Engagement

					When he was explaining that electric and magnetic fields will actually increase and decrease the magnitude with respect to the position with respect to space, but they may not change with respect to time, I was listening to him and actually I was very curious and i wanted to understand the diagram on the slide because it looks like a DNA structure, he started explaining the diagram, I was very happy, I was trying to align whatever he was trying to say, this part was actually very good for me		
			245 - 425	What has happened during this video segment	first of all i felt that, he was using too much of text on slide and he just reading the content, I was just listening, because i was also looking at the slide at same time	Passive Watching	Disengagement

		V2	125 - 700	What has happened during this video segment	I liked the way the presenter uses the transition and I liked the way he vanishes. Maybe I like visualizing things. So that is the reason when there is a demonstration, I'm very interested.	Passive Watching	Disengagement
				After listening to dumb hole concept, what was your state?	But I can't say that I was truly connected. I could not connect much. I can see that this thing's what he's saying. I just remember	Disconnection	Disengagement
		V3	100 - 305	So what was happening right before this point?	Because of the sound. It was lounder for me, I was getting a little disturbed. not always but sometimes it is high	Disconnection	Disengagement
2	P2	V1	99 - 250	What was happening in this video? How did you find this part? Were you reading the text initially	This video was too contained, heavy and too abstract, content is very alien to me, I was reading more and listening	Passive Watching & Disconnection	Disengagement

			260 - 370	What has happened from this point?	this captured my attention this point because theory may generally when we look for why do we need this theory, So I was trying to connect this structure with my existing knowledge of the mathematical theorems	Attention & Recall	Engagement
		V2	125 - 700	What was happening in this video? Was it interesting for you?	I didn't pay attention, I don't think I got any content	Passive Watching	Disengagement
		V3	99 - 385	What has happened in this video, have you watched this movie?	I have watched this movie, but within this video clip I don't think I was able to understand it . But I didn't get the connection with the clip itself, But there was no connection	Disconnectio n	Disengagement

3	P3	V1	100 - 429	What was happening during this whole video?	From here, I started losing my concentration because I was unfamiliar with those experiments. So I was thinking, what is that experiment?. I never heard about this. So that is why I think I lost my Interest.	Disconnection	Disengagement
					For example, whenever he was talking about Einstein's theory of relativity, I was somewhere. But completely I was lost. I was losing my interest because all the things were new for me and nothing was going inside my mind.	Aversion	Disengagement

					There were no relevant examples and things were written on the board, which was like, oh, there is something which is important. I lose my interest. Okay, what is this? What is this? I kept on reading that content, and also I was listening. So that is why I think I lost my interest. So where did you start reading that content? When he was pointing out to the board. So I read, that can be important information. But he kept on speaking, so I lost my interest.	Aversion	Disengagement
		V2	120 - 160	Was it not interesting for you?	Actually, it was interesting for me because black holes are still something which I'm not familiar with exactly.	Curiosity	Engagement

			165 - 700	Is there anything you want to share about this video segment	I'm not familiar with exactly what it is. So he was explaining black hole and then he moved to wormhole, then dumb hole, I have already mentioned there were so many new terms. but because of those animations, I was able to watch the video	Passive Watching	Disengagement
					I was listening to him, he talked about black holes, then he talked about singularity. Then he talked about dumb hole. Now he talked about wormholes. I was not connecting at that time	Passive Watching	Disengagement
					Then there was not a very high cognitive load. But yes, I was thinking because of videos, I was able to recollect the terms, whatever he was saying.	Passive Watching	Disengagement

		V3	103 - 180	What was happening during this part of the video? Have you watched the Interstellar movie?	I have watched the movie, but there was some confusion in my mind. But I thought this clip was from the movie Inception.	Aversion	Disengagement
			235 - 260	Were you listening to the conversation ? What was your state during this segment?	I was also trying to connect this with the previous video. Few terms discussed in previous videos have been used in this video, they are talking about wormholes or that is black holes. So I was like connecting again, So it was very much interesting for me and second thing, he was explaining some concept. Again, that was new for me, how they can escape from there. So that made me interesting	Curiosity	Engagement

			325 - 380	What was happening during this part of the video?	In this part, they are not talking about anything. They are just moving simulations. So I don't think cognitive engagement was very high in this part	Passive Watching	Disengagement
4	P4	V1	100 - 429	What was the reason for low rating on likert scale ?, what was your state in this video? Would you like to share your experience about the video?	I was not concentrating much, i was just overlooking, I was not comfortable with english, It was mostly boredom, I was thinking about some other things rather than concentrating	Aversion, Passive Watching	Disengagement
		V2	115 - 530	What was your state in this segment ?	I was distracted and felt monotonous, unable to understand anything, tried to understand but could not	Aversion, Passive Watching	Disengagement
				Were you able to comprehend the concepts	I was just looking at the animations	Passive Watching	Disengagement

				and animation			
			550 - 650	Do you remember this example? What has happened during this part of the video?	I thought, let's concentrate again,I was trying to focus, I understood the concept of blackhole, wormhole and dumb hole	Attention	Engagement
		V3	125 - 225	Did you like this part?, what was your state?, were you engaged or disengaged?	Initially i tried understanding, but couldn't understand due the accent , I was not able to comprehend the conversation	Passive Watching	Disengagement
			240 - 280	What has happened during this part of the video?	I was curious to understand how they will implement the plan to reach the miller planet, there was a supsense what will happen next ?	Curiosity	Engagement

5	P5	V1	100 - 429	What was your state while watching this video, what the reason for your rating in likert scale for cognitive and affective engagement	all the time I was kind of thinking and going back to the prior knowledge that I have about physics. And these experiments, also, I've read like Michelson Morley experiment and Maxwell's theory of electromagnetism. I'm also aware, so I was just kind of going back. I was trying to connect, like what I remembered, and I remembered a large chunk of it	Recall & Active Watching	Engagement

		V2	116 - 125	What has happened during this video segment?	there was astronomy, we studied one course on astronomy, and that's where these terms kind of came up. The math was not there at that time also, and I remembered that Schwarzschild radius is something that I've gotten before and here it was kind of mentioned again, and it was just a kind of refinement. Because I didn't remember what the Schwarzschild radius actually was. So here he just explained again, which is good. And yeah, so I could recall what it actually was about	Recall	Engagement

			205 - 330	What was your state while watching this video?	Because, I have encountered and read all of this stuff earlier, Here, I was more or less aware that what he's talking about, and maybe the variable, the terminology, which he has used in this part, So it was not something I was focusing too much on, because I had already kind of had studied and read about this earlier	Passive Watching	Disengagement

			330 - 380	Explain what was happening here?	this was the kind of the newest part like that I encountered in the video, because I don't recall myself at that time. Also, I didn't recall myself anything knowing anything about photon sphere, I knew that there was the there is this phenomena that light comes across and goes back to your head and but I didn't recall or the hearing about photons sphere or something. So I was kind of listening to him, it was a new term.	Curiosity	Engagement

			500 - 600		So I was trying to also predict what could happen. And I knew that there is a term called spaghettification, which happens after the event horizon, but I think I thought that happened here, but here, a new phenomena, which I was not aware of. So I was kind of also with him predicting like, and this is a common example which is used for explaining wormhole	Curiosity	Engagement
					when you travel at the speed of light towards is the most interesting part	Curiosity	Engagement
		V3	100 - 200	Were you engaged with the movie clip?	I was not like focusing on physics because I kind of knew that it's kind of already in it because it's sci fi, i was known throughout the video I would say I was not very engaged	Passive Watching	Disengagement

			205 - 260	Was there any change during this part, answer relatively with previous clip	I was more cognitively engaged because they were talking about the singularity event horizon and this visual of black hole, I was trying to relate it back to the first video and other photos of simulations of black holes that I've seen, okay, and I was just kind of trying to relate how it actually looks like and how it's being shown here	Curiosity	Engagement
			280 - 375	What about the last part of this clip?	I was not cognitively engaged, So I was more of seeing VFX this from just not thinking too much	Active Watching	Engagement

6	P6	V1	100 - 150	Could you explain what was your state during this section of the video ?	So I thought of where I heard about the Michaelson Morley experiment. I studied it in my first year of photonics . Just recalled that part, actually. I remember the experiment and results and diagram from your graduation. Basically, before engineering, I joined a course called photonics course it was a like Basic physics	Recall	Engagement

			160 - 595	Were you engaged with the content ?	I understood everything because I was familiar with most of it. But somehow I never remember connecting these two, actually, the special relativity theory and Michaelson Morley experiment, even though that is how it should be. I think maybe because I studied it during that time, I did not make that connection, but independently, I knew it	Disconnection	Disengagement

				Are you attentive during this part of the video ?	I was just Listening to the standing waves. I know about it, postulates also. I know, but, yeah, I was reading a circle over there. You are reading the text, which is on the moon. Okay. And when he was saying something else, also, I was reading some other area, because even not really concentrating on whatever he was saying, because anyway, he already said no, so there was more time to actually read	Aversion	Disengagement
			495 - 595	What has happened during the section ?	I noticed that. I paid attention. I noticed that it was an important thing to pay attention	Attention	Engagement
		V2	130 - 205	Till here it is the first part, what was your state in this section ?	So I remember, like, I don't remember the specifics, but I've heard of these things, so it was like I was trying to recall. Recall, yeah	Recall	Engagement

			210 - 360	Can you explain what was was happening here?, was it same like previous part, was it different in this part	Cognitive load, I think, was very minimal because it was okay. Very easy. It did not felt challenging	Passive Watching	Disengagement
			375 - 700	What has happened during the section ?	But this point was interesting. I wrote in the survey form, This was also an important point. I remember the cosmological concept, they were interesting points. But this concept particularly was interesting. Now I remember. Dumb hole, It was something that was made in Lab	Curiosity	Engagement

		V3	103 - 275	Were you listening to the conversation in movie clip	I don't remember actually, Rest of it, man, that's what in the movie particularly I don't remember anything actually because I think I was just watching. It's not particularly attending to anything specific	Passive Watching	Disengagement
			280 - 355	Any peculiar thing which you want to share about last part of the video	Sound was loud, I think	Sound	Media
7	P7	V1	105 - 290	What was happening during this period?Do you remember these concepts?	Listening to him, I was not focusing more on the text. Just looking at that diagram, I only read it when he pointed it	Passive Watching	Disengagement
					I can't remember actually even now, I lost thought here, I don't recall, I just forgot	Disconnection	Disengagement

			300 - 375	Was it is Interesting for you ? Was it boring ? tell me what has happened after this point	He was talking about inertial reference frames, that was Interesting, later he was explaining about postulates, I could relate, I could understand	Active Watching	Engagement
		V2	120 - 155	What was your state during this video segment?	Schwarzschild radius is a new concept and new term for me. I was excited for whatever was coming	Curiosity	Engagement
			160 - 560		Not that interesting, gravitational lensing and the photon sphere. It was specifically about light. So I was like, not that interested	Passive Watching	Disengagement
				Do you like the animations used in this video segment?	I don't love them, its okay for reference		
				Were you judging those animations?	I was judging those animations, it could have been better		

				Here he is explaining to you a theory which you already knew. So what was your state?	I was still enjoying it. It was to refine the whole thing. I don't actively think about it and at dumb hole topic I lost my interest	Disconnection	Disengagement
		V3	105 - 180	Have you watched the movie, do you remember this scene?, were you attentive during this part of the video?	I don't remember this scene, That's kind of boring, from the beginning to this point as one section, I was moderately engaged	Passive Watching	Disengagement
			190 - 265	What was happening here?	Scene change. I was shocked. Why did this happen? Is there any cut scene ? I was like, did they skip some portion of the movie or was it like this only that's why I was thought of shocked?, probably I don't remember	Curiosity	Engagement

Section III : Reference Survey Form

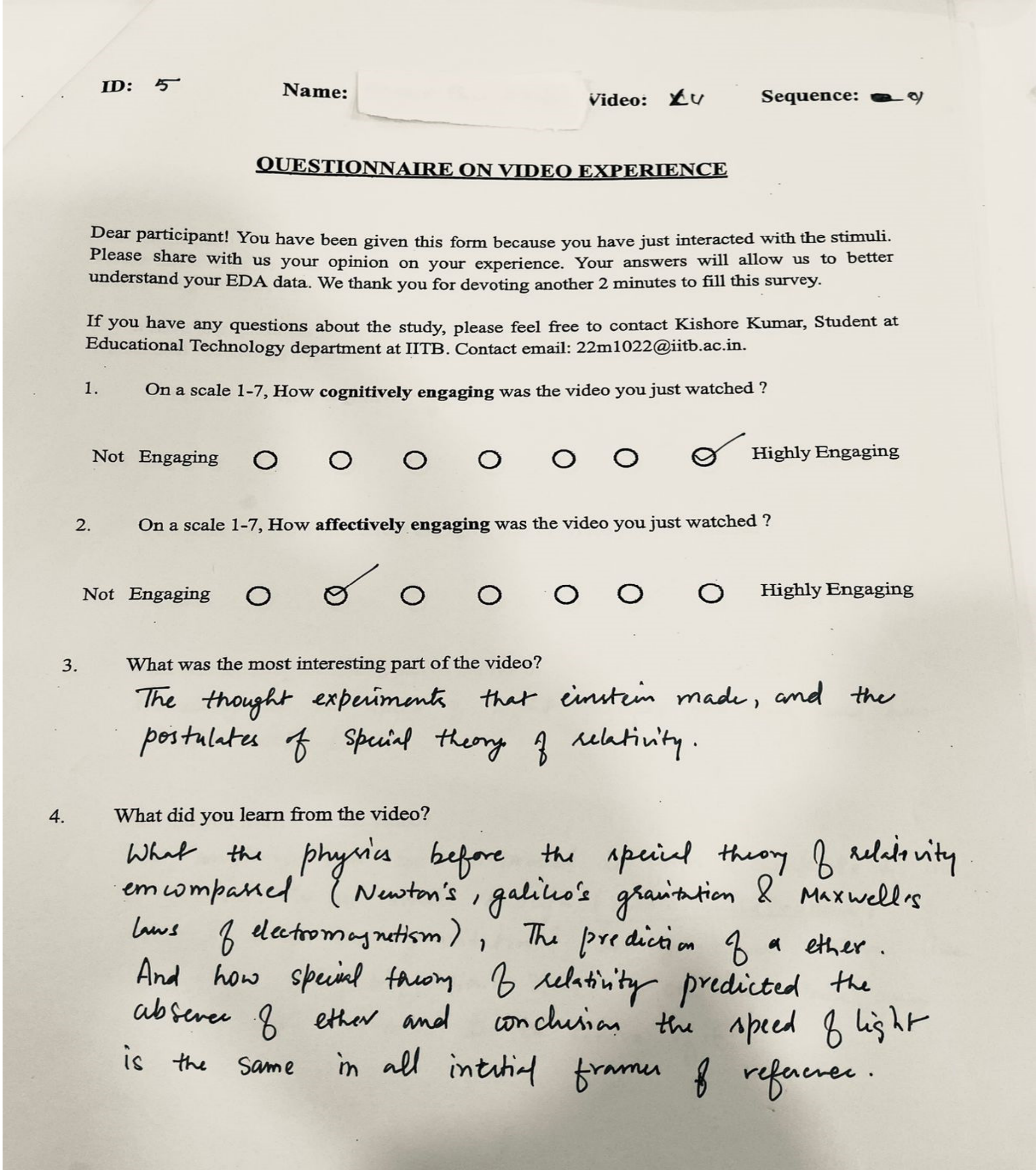

ID: 5 **Name:** [illegible] **Video:** [illegible] **Sequence:** [illegible]

QUESTIONNAIRE ON VIDEO EXPERIENCE

Dear participant! You have been given this form because you have just interacted with the stimuli. Please share with us your opinion on your experience. Your answers will allow us to better understand your EDA data. We thank you for devoting another 2 minutes to fill this survey.

If you have any questions about the study, please feel free to contact Kishore Kumar, Student at Educational Technology department at IITB. Contact email: 22m1022@iitb.ac.in.

1. On a scale 1-7, How **cognitively engaging** was the video you just watched ?

Not Engaging ○ ○ ○ ○ ○ ○ ✓ Highly Engaging

2. On a scale 1-7, How **affectively engaging** was the video you just watched ?

Not Engaging ○ ✓ ○ ○ ○ ○ ○ Highly Engaging

3. What was the most interesting part of the video?

The thought experiments that einstein made, and the postulates of special theory of relativity.

4. What did you learn from the video?

What the physics before the special theory of relativity encompassed (Newton's, galileo's gravitation & Maxwell's laws of electromagnetism), The prediction of a ether. And how special theory of relativity predicted the absence of ether and conclusion the speed of light is the same in all inertial frames of reference.
